# Generation of Aurachin Derivatives by Whole-Cell Biotransformation and Evaluation of Their Antiprotozoal Properties

**DOI:** 10.3390/molecules28031066

**Published:** 2023-01-20

**Authors:** Sebastian Kruth, Cindy J.-M. Zimmermann, Katharina Kuhr, Wolf Hiller, Stephan Lütz, Jörg Pietruszka, Marcel Kaiser, Markus Nett

**Affiliations:** 1 Laboratory of Technical Biology, Department of Biochemical and Chemical Engineering, TU Dortmund University, 44227 Dortmund, Germany; 2 Institute of Bioorganic Chemistry, Heinrich Heine University Düsseldorf at Forschungszentrum Jülich and Bioeconomy Science Center (BioSC), 52426 Jülich, Germany; 3 NMR Laboratory, Department of Chemistry and Chemical Biology, TU Dortmund University, 44227 Dortmund, Germany; 4 Laboratory of Bioprocess Engineering, Department of Biochemical and Chemical Engineering, TU Dortmund University, 44227 Dortmund, Germany; 5 Institute of Bio- and Geosciences: Biotechnology (IBG-1), Forschungszentrum Jülich, 52428 Jülich, Germany; 6 Swiss Tropical and Public Health Institute, 4123 Allschwil, Switzerland; 7 Swiss Tropical and Public Health Institute, University of Basel, Petersplatz 1, 4002 Basel, Switzerland

**Keywords:** antiprotozoal, aurachin, biotransformation, *Escherichia coli*, quinoline, quinolone

## Abstract

The natural product aurachin D is a farnesylated quinolone alkaloid, which is known to possess activity against the causative agent of malaria, *Plasmodium* spp. In this study, we show that aurachin D inhibits other parasitic protozoa as well. While aurachin D had only a modest effect on *Trypanosoma brucei rhodesiense*, two other trypanosomatids, *T. cruzi* and *Leishmania donovani*, were killed at low micromolar and nanomolar concentrations, respectively, in an in vitro assay. The determined IC_50_ values of aurachin D were even lower than those of the reference drugs benznidazole and miltefosine. Due to these promising results, we set out to explore the impact of structural modifications on the bioactivity of this natural product. In order to generate aurachin D derivatives with varying substituents at the C-2, C-6 and C-7 position of the quinolone ring system, we resorted to whole-cell biotransformation using a recombinant *Escherichia coli* strain capable of aurachin-type prenylations. Quinolone precursor molecules featuring methyl, methoxy and halogen groups were fed to this *E. coli* strain, which converted the substrates into the desired analogs. None of the generated derivatives exhibited improved antiprotozoal properties in comparison to aurachin D. Obviously, the naturally occurring aurachin D features already a privileged structure, especially for the inhibition of the causative agent of visceral leishmaniasis.

## 1. Introduction

Many parasitic protozoa are found in tropical and subtropical areas, where they cause severe, often lethal human diseases [1]. Well-known are infections with *Plasmodium* spp., which are transmitted by mosquito bites. The parasites colonize and eventually destroy red blood cells, resulting in malaria [2]. Although malaria remains a scourge of humanity, a number of medications are available for its prevention as well as for suppressing its progression [2]. For several other protozoa-associated diseases, in particular those classified as neglected tropical diseases (NTDs) [3], there are fewer therapeutic options. Examples are African sleeping sickness, Chagas disease, and visceral leishmaniasis, which result from infections with trypanosomatids, namely *Trypanosoma brucei*, *T. cruzi* and *Leishmania donovani* [4,5,6]. The currently approved trypanocidal drugs exhibit severe side effects, which limit their use [7]. On the other hand, African sleeping sickness and visceral leishmaniasis are fatal if left untreated. This dilemma illustrates the urgent need for new antiprotozoal drugs with improved toxicity profiles.

**Figure 1:**
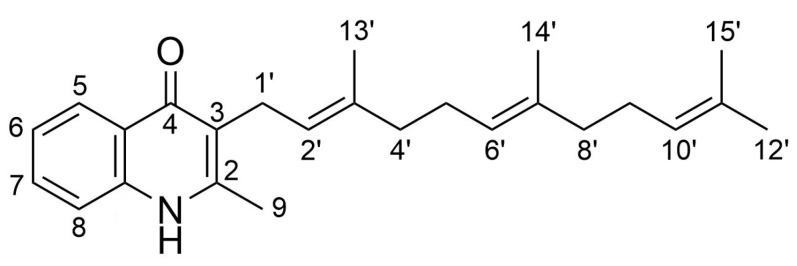
Chemical structure of aurachin D (**1**).

Natural products represent a promising source for the discovery of drugs against infectious diseases, including those caused by protozoa [8]. An illustrative example is the quinoline alkaloid quinine, which was first isolated from the bark of a *Cinchona* tree [9]. Quinine interferes with the ability of the malarial pathogen to detoxify hemoglobin-derived metabolites. Furthermore, it served as a lead structure for the development of several synthetic antimalarial drugs featuring a quinoline scaffold, such as chloroquine [9]. In recent years, quinolines and quinolones have gained increasing attention as trypanocidal agents [10]. In this study, we set out to assess the activity of aurachin D against human infective forms of pathogenic trypanosomatids and malaria. Aurachin D (Figure 1, **1**) belongs to a family of bacterial quinolone antibiotics, of which some members, including **1**, were already demonstrated to possess antiprotozoal properties [11,12]. However, previous studies only included *Plasmodium falciparum* and *T. brucei gambiense* as test organisms. It was hence unclear whether these natural products would also be active against other protozoa. For us, **1** was an attractive test compound, because we had previously established a scalable process for its biocatalytic production using recombinant *E. coli* cells [13]. The production of **1** in a heterologous host eliminated the need for extensive downstream processing since the biosynthesis could be efficiently directed to the target molecule. Subsequent biosynthetic modifications are excluded, thereby circumventing complex chromatographic separations of byproducts [14]. Moreover, we expected that the described method for the production of **1** would also allow the generation of structural analogs by means of whole-cell biotransformation [15].

Here, we report the results of our in vitro testing, which revealed **1** as an extremely potent antiprotozoal agent, even in comparison with approved reference drugs. Due to this promising outcome, we subsequently generated several derivatives of **1** by feeding the aurachin-producing *E. coli* strain with appropriate precursor molecules. The resulting compounds were subjected to bioactivity testing following their purification. In this way, the impact of structural modifications on the antiprotozoal properties of aurachins could be explored.

## 2. Results

For in vitro bioactivity testing, **1** was recombinantly produced from 2-methyl-1*H*-quinolin-4-one with an *E. coli* strain expressing the aurachin farnesyltransferase AuaA [13,16]. The compound was purified by HPLC, and its purity was determined to be >95% by ^1^H NMR analysis. Subsequently, **1** was assayed against *P. falciparum* NF54, *T. brucei rhodesiense* STIB 900, *T. cruzi* Tulahuen C4 and *L. donovani* MHOM-ET-67/L82, using the approved drugs chloroquine, melarsoprol, benznidazole, and miltefosine as positive controls. Consistent with previous literature reports [11,12], **1** showed a potent antiplasmodial effect with an IC_50_ value of 0.012 μM against *P. falciparum*. In addition, we observed significant trypanocidal activities. The IC_50_ values of **1** against *T. brucei rhodesiense* and *T. cruzi* were in the lower micromolar range, i.e., 4.5 μM and 1.3 μM, respectively. In the case of *L. donovani*, **1** was even active at nanomolar concentrations (IC_50_ 0.044 μM). It is noteworthy that **1** exhibited lower IC_50_ values than the reference drugs benznidazole (IC_50_ 2.5 μM against *T. cruzi*) and miltefosine (IC_50_ 0.732 μM against *L. donovani*). Since the aurachins, including **1**, are known to inhibit electron transport processes in the respiratory chain not only of prokaryotic but also of eukaryotic organisms [17,18], it was necessary to evaluate possible toxic effects on mammalian cells. For this, we used rat skeletal myoblasts, and we determined an IC_50_ value of 0.131 mM for **1**, which corresponds to a selectivity index of 2970 for the antileishmanial effect. Overall, this indicates a favorable window between antiprotozoal activity and toxicity. Due to these promising results, we set out to explore the impact of structural modifications on the bioactivity of **1**. Although one study had already substantiated the importance of the farnesyl side-chain for antiplasmodial activity [12], the effects of substitutions on the quinolone nucleus had not been systematically interrogated. Recent investigations of structure–activity relationships (SAR) of aurachin-type compounds focused exclusively on their inhibitory effect on bacterial cytochrome *bd* oxidases [19,20]. It was hence questionable whether the SAR data reported in these studies would also be applicable to antiprotozoal activities.

### 2.1. Generation of Aryl-Substituted Aurachin D Analogs by Whole-Cell Biotransformation

In contrast to previous SAR studies, which relied on total synthesis for the preparation of aurachin derivatives [12,19,20], we decided to pursue a hybrid strategy involving the biocatalytic conversion of synthetically generated quinolone precursor molecules [21]. The latter approach became a feasible option after the construction of an *E. coli* strain capable of regioselectively introducing a farnesyl moiety at C-3 of 2-methyl-1*H*-quinolin-4-one [13]. The substrates for the biotransformation experiments were prepared in a two-step sequence from ethyl acetoacetate and substituted aniline derivatives (Scheme 1, Figures S1−S17).

Previous in vitro studies already indicated that AuaA tolerates diverse substituents at C-2, C-6, or C-7 of a 1*H*-quinolin-4-one substrate, whereas the enzyme is much less permissive concerning variations in other positions [16]. This regiopreference was considered in the substrate selection. The generated compounds as well as four commercial substrates, 1*H*-quinolin-4-one, 2,6-dimethyl-1*H*-quinolin-4-one, 7-methoxy-2-methyl-1*H*-quinolin-4-one and 6-fluoro-2-methyl-1*H*-quinolin-4-one, were added to growing cultures of the *auaA*-expressing *E. coli* strain [13] to give a final substrate concentration of 10 or 20 mg/L. Afterward, the cultivation was resumed for 24 h before the biotransformation products were recovered with an adsorber resin and purified by HPLC. Molar yield coefficients (Y_P/S_) were determined post-purification and then used to calculate the substrate conversion in relation to 2-methyl-1*H*-quinolin-4-one, which is farnesylated by AuaA to **1**. Although no replicates were included in this analysis, the resulting values allow a cautious estimation of the efficiency of the whole-cell biotransformation. Overall, we observed satisfactory conversion (Table 1). Only three out of 12 tested unnatural substrates could not be converted into aurachins. While hexyl and nitro substituents are likely too bulky to be tolerated by AuaA, the installation of methoxy groups seems to be less efficient. The two tested monomethoxylated substrates were converted, albeit at a comparatively low level, whereas the quinolone precursor featuring two methoxy groups at C-6 and C-7 was not accepted. Interestingly, some substrates, such as 2,6-dimethyl-1*H*-quinolin-4-one and 7-chloro-2-methyl-1*H*-quinolin-4-one were converted much more efficiently into aurachins than anticipated from an in vitro analysis of AuaA [16]. This might be explained by the increased lipophilicity of these compounds in comparison to 2-methyl-1*H*-quinolin-4-one, which could have positively influenced their cellular uptake. The reason for the good conversion of 1*H*-quinolin-4-one under in vivo conditions is, however, unclear.

### 2.2. Spectroscopic Analysis of the Generated Aurachin D Analogs

The identities of the aurachin analogs were confirmed by comparison of their spectroscopic data (Figures S18–S43 in Supplementary Materials) with literature values [12,16,20]. Two of the produced compounds, **4** and **10**, have not been reported before and were therefore subjected to a comprehensive characterization.

**Figure 2:**
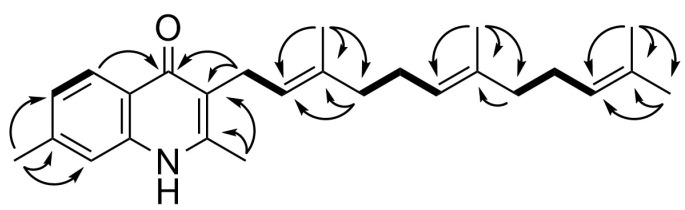
Selected 2D NMR correlations in **4**.

The high-resolution (HR) ESI-MS spectrum of **4** showed a pseudomolecular ion peak at *m/z* 378.2729 [M+H]^+^, which was assigned to an empirical formula of C_26_H_35_NO and 10 double bond equivalents. An analysis of the ^1^H NMR spectrum revealed three resonances in the aromatic region. The splitting of these signals and the concomitant coupling constants were consistent with an ABX spin system, which could be assigned to a 2,3,7-trisubstituted 4-quinolone moiety following the interpretation of ^13^C NMR, HSQC and HMBC data. The two methyl substituents at C-2 and C-7 were deduced from correlations observed in the HMBC spectrum (Figure 2). The farnesyl residue of **4** was identified on the basis of COSY and HMBC data. Its connection with C-3 concluded the structure determination.

The HR ESI-MS spectrum of compound **10** exhibited two distinct [M+H]^+^ ion peaks at *m/z* 442.1668 and *m/z* 444.1652 with almost equal intensities. This isotopic pattern is characteristic of a compound featuring a single bromine atom. The molecular formula of **10** was established as C_25_H_32_NOBr. It accounts for 10 double bond equivalents. The NMR spectra of **10** were very similar to those of **4**, except that the resonance for the methyl group at C-7 was missing. Furthermore, several signals in the aromatic region were shifted. Interpretation of HMBC data led to the conclusion that **10** possesses a 2,3,6-trisubstituted 4-quinolone motif. A methyl group is located at C-2 and a farnesyl side chain at C-3 in accordance with **4**. The bromine atom, which was deduced from the MS analysis, was assigned to C-6. Its electron-withdrawing, negative-inductive effect explains the downfield shifts of the protons at C-5 and C-7, respectively.

### 2.3. Evaluation of Antiprotozoal Properties

Serial dilution assays with 11 dilution steps in 96-well plates were used in order to determine the IC_50_ values of the generated aurachin D analogs against *P. falciparum* NF54 (erythrocytic stages), *T. brucei* rhodesiense STIB 900 (bloodstream forms), *T. cruzi* Tulahuen C4 (intracellular amastigotes) and *L. donovani* MHOM-ET-67/L82 (axenic amastigotes). Potential cytotoxic effects were evaluated with L6 myoblast cells (Table 2).

The bioactivity testing revealed that the antiprotozoal properties of aurachin D can be significantly altered by furnishing its quinolone backbone with different functional groups. Of particular interest was the observation that these structural modifications have unequal effects on the tested protozoa. An illustrative example is **4**, the 7-methyl derivative of **1**, which showed comparable antiplasmodial activities to the parental compound (IC_50_ 0.007 μM vs. 0.012 μM). On the other hand, **4** was the least active compound in our assay against the two *Trypanosoma* species. Another example concerns the 6-fluoro and the 6-chloro aurachin D analog. Both **7** and **8** exhibited comparable bioactivity on *T. brucei rhodesiense* and also comparable cytotoxicity, as evidenced by their IC_50_ values against L6 cells. In contrast, their potency against *T. cruzi* and *L. donovani* was antipodal. Two conclusions can be drawn from these results: (i) It is possible to tune the antiprotozoal activity range of aurachins through minor structural variations; (ii) The lack of consistent SAR trends suggests that the aurachins exert their effects via different targets in the tested protozoa and also in mammalian cells.

If the IC_50_ values of **1–10** are compared to reference drugs, it becomes evident that the aurachins exhibit only moderate activities against *T. brucei rhodesiense*, the causative agent of *East African* trypanosomiasis (sleeping sickness). Much more interesting are the inhibitory effects on *T. cruzi* and *L. donovani*. As stated before, **1** exhibits competitive IC_50_ values and also a favorable selectivity index. These properties could not be further improved with the structural modifications introduced in this project. A comparison of their IC_50_ values shows that **2** and **7** were the two most active trypanocidal agents of all aurachin D derivatives tested (Table 2). However, since **2** was found to be about six-fold more toxic for L6 cells, its selectivity index significantly decreased. Further studies are now necessary to identify the target of **1** and **7** in *T. cruzi* and *L. donovani* as well as to evaluate their in vivo efficacies. Moreover, the validation of the antileishmanial activity is reasonable in view of the limitations of axenic amastigote models [22].

## 3. Discussion

NTDs caused by protozoa are affecting millions of people worldwide. Because of the ongoing climate change, which may impact the distribution of NTDs, and the emergence of drug-resistant parasites, new antiprotozoal drugs are urgently needed [7,23]. Natural products are an essential resource for drug development. Previous studies have already unveiled the aurachin scaffold as a suitable starting point for antimalarial drug development [11,12]. Our extended testing shows that aurachin D is also highly active against other pathogenic protozoa like *T. cruzi* and *L. donovani*. In order to explore the effects of structural modifications on its bioactivity, we decided to produce aryl-substituted aurachin D analogs by whole-cell biotransformation. This strategy merges the versatility of organic synthesis with the efficiency of biocatalysis for generating natural product analogs from chemically synthesized precursor molecules, as recently demonstrated for the generation of novel chalanilines [24], physostigmines [25] and amicetins [26], respectively. In the present study, a total of nine aryl-substituted aurachin D analogs were produced after feeding a recombinant *E. coli* strain with various 1*H*-quinolin-4-ones.

Interestingly, even substrates featuring large substituents like bromine were successfully integrated into aurachin biosynthesis. Except for compounds **2** and **4**, which had lower IC_50_ values against *P. falciparum* than aurachin D, none of the generated derivatives showed increased activities against the tested protozoa. However, the antiprotozoal activity range of aurachin D could be significantly altered through minor structural variation. We assume that aurachin D can be biotransformed even further by recruiting additional enzymes from aurachin biosynthesis [27]. This will make it possible to access an even larger chemical space.

## 4. Materials and Methods

### 4.1. General Experimental Procedures

All chemicals and solvents were obtained from commercial sources and used without further purification, if not otherwise mentioned. Chemical syntheses were performed under inert conditions in a nitrogen atmosphere via Schlenk technique. TLC was performed on TLC-foil Polygram SIL G/UV_254_ (Macherey-Nagel). For the staining of TLC plates, ceric molybdate solution (12.5 g phosphomolybdic acid hydrate, 5.0 g ceric(IV) sulfate tetrahydrate, 30 mL conc. sulfuric acid, 200 mL demineralized water) and ninhydrin stain (400 mg ninhydrin, 200 mL ethanol) were used followed by heating with a heat gun. NMR spectra of 1*H*-quinolin-4-ones were recorded on a Bruker Avance/DRX 66. NMR analyses of aurachins were carried out at ambient temperature using either a Bruker AV 700 Avance III HD or a Bruker AV 600 Avance III HD spectrometer, both of which were equipped with a 5 mm helium-cooled inverse quadruple resonance cryoprobe. For NMR measurements, all compounds (except for **Yh**) were dissolved in deuterated methanol (methanol-*d*_4_), which also served as internal standard to calibrate spectra to δ_H_ = 3.31 ppm and δ_C_ = 49.0 ppm. Compound **Yh** was dissolved in deuterated dimethyl sulfoxide (DMSO-*d*_6_), and the spectra were calibrated to δ_H_ = 2.50 ppm and δ_C_ = 39.5 ppm. High-resolution ESI-MS spectra were recorded in positive ionization mode on an Agilent 1260 Infinity HPLC system combined with a Bruker Daltonics Compact quadrupole time of flight mass spectrometer. The HPLC was operated with a Nucleoshell RP 18 ec column (100 × 2 mm, 2.7 μm; Macherey-Nagel) at the following conditions: Flow rate: 0.4 mL/min. Column oven: 40 °C. Mobile phases: acetonitrile and water with 0.1% (*v/v*) formic acid. Gradient: 0–10 min: 2–98% ACN; 10–15 min: 98% ACN; 15–17 min: 98–5% ACN; 17–20 min: 5% ACN; 20–27 min: 5–100% ACN; 27–28 min: 100–30% ACN; 28–32 min: 30% ACN. For the MS analyses, a capillary voltage of 4.5 kV, a desolvation gas (N_2_) temperature of 220 °C and a dry gas (N_2_) flow rate of 12 L/min were used.

### 4.2. Synthesis of 1H-Quinolin-4-One Substrates

For the preparation of 1*H*-quinolin-4-ones **Ya**, **Yc** and **Ye-h**, a previously described protocol [28] was adapted. The aniline derivative (10 mmol, 1 equiv.) was dissolved in 20 mL ethanol under a nitrogen atmosphere. Ethyl acetoacetate (1.9 mL, 15 mmol, 1.5 equiv.), Zn(ClO_4_)_2_·6H_2_O (373 mg, 1 mmol, 0.1 equiv.) and anhydrous MgSO_4_ (1.20 g, 10 mmol, 1 equiv.) were added and the reaction mixture was stirred at room temperature for 24–48 h. The reaction was monitored via TLC until equilibrium was reached. The reaction mixture was filtered through a pad of celite, and the filter cake was washed with dichloromethane. The solvent was evaporated under reduced pressure. The formed enamine was rapidly added to 200 mL degassed diphenylether. Activated molecular sieve powder 4Å (1 g) was added. The reaction mixture was stirred at 250 °C for 1 h. The reaction mixture was cooled down to room temperature, and the precipitate formed was recovered by filtration. The filter cake, which consisted of product and molecular sieve, was washed with petroleum ether. Subsequently, methanol was added to the filter cake in order to separate the methanol-soluble product from the molecular sieve by filtration. The solvent was evaporated under reduced pressure. Column chromatography was performed using silica gel (0.040–0.063 mm; Merck) as stationary phase and a mixture of dichloromethane and methanol (9:1) as eluent. For the separation of regioisomers **Ye** and **Ye’**, a preparative HPLC was operated with a HyperClone ODS (C18) column (250 × 10 mm, 5 µm) at the following conditions: Flow rate: 5 mL/min. Column oven: 25 °C. Mobile phases: methanol and water. Gradient: 0–2 min: 10% MeOH; 2–3.7 min: 10–20% MeOH; 3.7–25 min: 20% MeOH; 25–26.2 min: 20–40% MeOH; 26.2.-45 min: 40% MeOH; 45–46.2 min: 40–10% MeOH; 46.2–60 min: 10% MeOH.

For the preparation of 1*H*-quinolin-4-ones **Yb** and **Yd**, a previously described procedure was adapted [29,30]. Acetoacetic acid (1 equiv.) was dissolved in anhydrous ethanol. Ceric(IV) ammonium nitrate (0.05 equiv.) and aniline derivative (1 equiv.) were added. After the mixture was stirred for 2–36 h at 25–40 °C, dichloromethane was added. The reaction mixture was washed with demineralized water and brine. The organic layer was dried with MgSO_4_, and the solvent was evaporated under reduced pressure. A CEM reaction tube with crude enamine dissolved in diphenyl ether was irradiated for 0.75–1.5 h at 190 °C with 280 W. Petroleum ether was added, and the precipitate was filtered and washed with petroleum ether and ethyl acetate. Column chromatography was performed using silica gel (0.040–0.063 mm; Merck) as stationary phase and a mixture of dichloromethane and methanol (9:1) as eluent.

### 4.3. Analytical Data of 1H-Quinolin-4-One Substrates

2,7-Dimethyl-1*H*-quinolin-4-one (**Ya**). ^1^H NMR (600 MHz, methanol-*d*_4_), δH [ppm] (*J* [Hz]) 2.45 (3 H, s, H-1‘), 2.48 (3 H, s, H-1′’), 6.17 (1 H, s, H-3), 7.22 (1 H, d,*J* 8.4, H-6), 7.33–7.30 (1 H, m, H-8), 8.08 (1 H, d, *J* 8.3, H-5). ^13^C NMR (150 MHz, methanol-*d*_4_), δC [ppm] 19.8 (C-1‘), 21.8 (C-1′’), 109.3 (C-3), 118.3 (C-8), 123.3 (C-4a), 125.9 (C-5), 126.9 (C-6), 141.8 (C-8a), 144.6 (C-7), 152.8 (C-2), 180.4 (C-4). HR-ESIMS: *m/z* 174.0916 [M+H]^+^ (calcd. for C_11_H_12_NO 174.0914; Δppm 1.1).

2,5-Dimethyl-1*H*-quinolin-4-one (**Ya’**). ^1^H NMR (600 MHz, methanol-*d*_4_), δH [ppm] (*J* [Hz]) 2.38 (3 H, s, H-1‘), 2.87 (3 H, s, H-1′’), 6.17 (1 H, s, H-3), 7.05 (1 H, d, *J* 7.2, H-6), 7.33–7.30 (1 H, m, H-8), 7.45 (1 H, dd, *J* 8.3, 7.2, H-7). ^13^C NMR (150 MHz, methanol-*d*_4_), δC [ppm] 19.3 (C-1‘), 23.8 (C-1‘‘), 111.2 (C-3), 117.0 (C-8), 124.1 (C-4a), 127.4 (C-6), 132.40 (C-7), 141.2 (C-5), 143.3 (C-8a), 151.0 (C-2), 183.1 (C-4). HR-ESIMS: *m/z* 174.0916 [M+H]^+^ (calcd. for C_11_H_12_NO 174.0914; Δppm 1.1).

6-Hexyl-2-methyl-1*H*-quinolin-4-one (**Yb**). ^1^H NMR (600 MHz, methanol-*d*_4_), δH [ppm] (*J* [Hz]) 0.92–0.87 (3 H, m, H-6‘‘), 1.40–1.29 (6 H, m, H-3‘‘, H-4‘‘, H-5‘‘), 1.68 (2 H, q, *J* 7.5, H-2‘‘), 2.46 (3 H, s, H-1‘), 2.75 (2 H, t, *J* 7.7, H-1‘‘), 6.19 (1 H, s, H-3), 7.47 (1 H, d, *J* 8.5, H-8), 7.54 (1 H, dd, *J* 8.5, *J* 2.0, H-7), 8.01 (1 H, d, *J* 2.0, H-5). ^13^C NMR (150 MHz, methanol-*d*_4_), δC [ppm] 14.4 (C-6‘‘), 19.8 (C-1‘), 23.7 (C-5), 30.0 (C-3‘‘), 32.6 (C-2‘‘), 32.9 (C-4), 36.6 (C-1‘‘), 109.4 (C-3), 119.0 (C-8), 124.6 (C-5), 125.3 (C-4a), 134.4 (C-7), 139.9 (C-8a), 140.2 (C-6), 152.5 (C-2), 180.4 (C-4). HR-ESIMS: *m/z* 244.1699 [M+H]^+^ (calcd. for C_16_H_22_NO_3_ 244.1696; Δppm 1.2).

6-Methoxy-2-methyl-1*H*-quinolin-4-one (**Yc**). ^1^H NMR (600 MHz, methanol-*d*_4_), δH [ppm] (*J* [Hz]) 2.46 (3 H, s, 3H, H-1‘), 3.89 (3 H, s, H-1′’), 6.19 (1 H, s, H-3), 7.33–7.30 (1 H, m, H-7), 7.49 (1 H, m, H-8), 7.63–7.61 (1 H, m, H-5). ^13^C NMR (150 MHz, methanol-*d*_4_), δC [ppm] 19.7 (C-1‘), 56.1 (C-1‘‘), 104.8 (C-5), 108.8 (C-3), 120.6 (C-8), 124.4 (C-7), 126.4 (C-4a), 136.3 (C-8a), 151.7 (C-2), 158.1 (C-6), 179.7 (C-4). HR-ESIMS: *m/z* 190.0866 [M+H]^+^ (calcd. for C_11_H_12_NO_2_ 190.0863; Δppm 1.6). Analytical data in agreement with those previously reported [31].

6,7-Dimethoxy-2-methyl-1*H*-quinolin-4-one (**Yd**). ^1^H NMR (600 MHz, methanol-*d*_4_), δH [ppm] (*J* [Hz]) 2.41 (3 H, s, H-1′), 3.90 (3 H, s, H-1′’), 3.92 (3 H, s, H-1′’’), 6.12 (1 H, s, H-3), 6.86 (1 H, s, H-8), 7.52 (1 H, s, H-5). ^13^C NMR (150 MHz, methanol-*d*_4_): δ [ppm] = 179.0 (C-4), 155.5 (C-7), 150.9 (C-2), 149.0 (C-6), 137.6 (C-8a), 119.2 (C-4a), 108.6 (C-3), 104.9 (C-5), 99.5 (C-8), 56.5 (C-1‘‘‘), 56.4 (C-1‘‘), 19.7 (C-1‘). HR-ESIMS: *m/z* 220.0971 [M+H]^+^ (calcd. for C_12_H_14_NO_3_ 220.0968; Δppm 1.4). Analytical data in agreement with those previously reported [32].

7-Chloro-2-methyl-1*H*-quinolin-4-one (**Ye**). ^1^H NMR (600 MHz, methanol-*d*_4_), δH [ppm] (*J* [Hz]) 2.44 (3 H, s, H-1‘), 6.17 (1 H, s, H-3), 7.34 (1 H, dd, *J* 8.7, *J* 1.9, H-6), 7.52 (1 H, d, *J* 1.9, H-8), 8.14 (1 H, d, *J* 8.7, H-5). ^13^C NMR (150 MHz, methanol-*d*_4_), δC [ppm] 19.9 (C-1‘), 110.1 (C-3), 118.3 (C-8), 123.9 (C-4a), 125.5 (C-6), 128.0 (C-5), 139.3 (C-7), 142.2 (C-8a), 153.5 (C-2), 180.0 (C-4). HR-ESIMS: *m/z* 194.0371 [M+H]^+^ (calcd. for C_10_H_9_ClNO 194.0373; Δppm 1.0). Analytical data in agreement with those previously reported [33].

5-Chloro-2-methyl-1*H*-quinolin-4-one (**Ye’**). ^1^H NMR (600 MHz, methanol-*d*_4_), δH [ppm] (*J* [Hz]) 2.39 (3 H, s, H-1‘), 6.11 (1 H, s, H-3), 7.29 (1 H, dd, *J* 7.6, *J* 1.2, H-6), 7.40 (1 H, dd, *J* 8.4, *J* 1.2, H-8), 7.49 (1 H, t, *J* 8.0, H-7). ^13^C NMR (150 MHz, methanol-*d*_4_), δC [ppm] 19.3 (C-1‘), 111.9 (C-3), 118.2 (C-8), 122.0 (C-4a), 127.6 (C-6), 132.7 (C-7), 134.3 (C-5), 144.1 (C-8a), 151.6 (C-2), 180.2 (C-4). HR-ESIMS: *m/z* 194.0371 [M+H]^+^ (calcd. for C_10_H_9_ClNO 194.0373; Δppm 1.0).

6-Chloro-2-methyl-1*H*-quinolin-4-one (**Yf**). ^1^H NMR (600 MHz, methanol-*d*_4_), δH [ppm] (*J* [Hz]) 2.46 (3 H, s, H-1‘), 6.20 (1 H, s, H-3), 7.53 (1 H, d, *J* 8.9, H-8), 7.65 (1 H, dd, *J* 8.9, *J* 2.3, H-7), 8.15 (1 H, d, *J* 2.3, H-5). ^13^C NMR (150 MHz, methanol-*d*_4_), δC [ppm] 19.9 (C-1‘), 109.9 (C-3), 121.0 (C-8), 125.2 (C-5), 126.4 (C-4a), 130.8 (C-6), 133.6 (C-7), 140.0 (C-8a), 153.4 (C-2), 179.3 (C-4). HR-ESIMS: *m/z* 194.0368 [M+H]^+^ (calcd. for C_10_H_9_ClNO 194.0373; Δppm 2.6). Analytical data in agreement with those previously reported [31].

6-Bromo-2-methyl-1*H*-quinolin-4-one (**Yg**). ^1^H NMR (600 MHz, methanol-*d*_4_), δH [ppm] (*J* [Hz]) 2.46 (3 H, s, H-1‘), 6.21 (1 H, s, H-3), 7.47 (1 H, d, *J* 8.8, H-8), 7.78 (1 H, dd, *J* 8.8, *J* 2.3, H-7), 8.32 (1 H, d, *J* 2.3, H-5). ^13^C NMR (150 MHz, methanol-*d*_4_), δC [ppm] 19.9 (C-1‘), 110.0 (C-3), 118.2 (C-6), 121.2 (C-8), 126.8 (C-4a), 128.5 (C-5), 136.3 (C-7), 140.4 (C-8a), 153.5 (C-2), 179.2 (C-4). HR-ESIMS: *m/z* 237.9865 [M+H]^+^ (calcd. for C_10_H_9_BrNO 237.9863; Δppm 0.8). Analytical data in agreement with those previously reported [34].

2-Methyl-6-nitro-1*H*-quinolin-4-one (**Yh**). ^1^H NMR (600 MHz, DMSO-*d*_6_), δH [ppm] (*J* [Hz]) 2.38 (3 H, s, H-1‘), 6.08 (2 H, s, H-3), 7.68 (2 H, d, *J* 9.1, 2H, H-8), 8.40 (2 H, dd, *J* 9.1, *J* 2.7, 2H, H-7), 8.80 (1 H, s, H-5). ^13^C NMR (150 MHz, DMSO-*d*_6_), δC [ppm] 19.5 (C-1‘), 109.9 (C-3), 119.6 (C-8), 121.5 (C-5), 123.5 (C-4a), 125.8 (C-7), 142.4 (C-6), 143.9 (C-8a), 151.4 (C-2), 176.2 (C-4). HR-ESIMS: *m/z* 205.0610 [M+H]^+^ (calcd. for C_10_H_9_N_2_O_3_ 205.0608; Δppm 1.0). Analytical data in agreement with those previously reported [35].

### 4.4. Whole-Cell Biotransformation and Product Recovery

The *E. coli* BL21(DE3) strain for the whole-cell biotransformation was taken from the strain collection of the Technical Biology group at TU Dortmund University and is available upon request. Cultures of this strain, which harbors the two plasmids pSK46 [13] and pJBEI-2997 [36], were grown in terrific broth (TB) medium [37] supplemented with 50 μg/mL kanamycin, 30 μg/mL chloramphenicol and 0.12% (*v/v*) glycerol. The cultivation was conducted at a 1 L scale in shaken Erlenmeyer flasks at 30 °C and 120 rpm. Substrates were added at the time of inoculation at a final concentration of 10 or 20 mg/L. Once the cultures had reached an optical density at 600 nm (OD_600_) of 1, they were induced by addition of 0.025 mM isopropyl *β*-d-1-thiogalactopyranoside (IPTG). The incubation was then continued for 24 h. At the end of fermentation, the adsorber resin XAD7HP was added to the cultures at a concentration of 2% (*w/v*). After 3 h of shaking at 90 rpm, the resin was separated from the culture broth by filtration and washed with water. Subsequently, the adsorbed compounds were eluted with 1 L methanol. The resulting extracts were dried with a rotary evaporator, resuspended in 60% methanol (100 mL) and extracted three times with dichloromethane (60 mL). The organic phases were collected and concentrated to dryness with a rotary evaporator. After resuspension in 1.5 mL methanol, the chromatographic purification was carried out using a Shimadzu HPLC system (LC-20 AD) equipped with a diode array detector (SPD-M20A) and a Nucleodur C18 Isis column (250 × 10 mm, 5 μm, Macherey-Nagel). For the separation, a linear gradient of acetonitrile in water supplemented with 0.1% (*v/v*) trifluoroacetic acid from 40% to 90% over 20 min was used. The gradient was kept at 90% for additional 7 min. The flow rate was set to 5.6 mL/min. In the case of **5** and **6**, a further purification step with the same column was required. For this, an isocratic separation with 55% acetonitrile in 45% water supplemented with 0.1% (*v/v*) trifluoroacetic acid was carried out. Except for 2-desmethyl aurachin D (**2**), which was recovered as a pale brownish oil after chromatography, all products were isolated as beige solids.

### 4.5. Spectroscopic Data of Aurachin Derivatives

2-Desmethyl aurachin D (**2**). ^1^H NMR (700 MHz, methanol-*d*_4_), δH [ppm] (*J* [Hz]) 1.54 (3 H, d, *J* 1.0, H-15′), 1.60 (3 H, d, *J* 1.3, H-14′), 1.62 (3 H, d, *J* 1.3, H-12′), 1.73 (3 H, d, *J* 1.3, H-13′), 1.94 (2 H, m, H-8′), 2.02 (2 H, m, H-9′), 2.10 (2 H, m, H-4′), 2.15 (2 H, m, H-5′), 3.32 (2 H, not resolved, H-1′), 5.04 (1 H, m, H-10′), 5.13 (1 H, m, H-6′), 5.38 (1 H, m, H-2′), 7.40 (1 H, ddd, *J* 1.2, 6.9, 8.4, H-6), 7.56 (1 H, dd, *J* 1.2, 8.4, H-8), 7.68 (1 H, ddd, *J* 1.5, 6.9, 8.4, H-7), 7.80 (1 H, s, H-2), 8.29 (1 H, dd, *J* 1.5, 8.4, H-5). ^13^C NMR (175 MHz, methanol-*d*_4_), δC [ppm] 16.1 (C-14′), 16.2 (C-13′), 17.7 (C-15′), 25.9 (C-12′), 26.7 (C-1′), 27.5 (C-5′), 27.7 (C-9′), 40.8 (C-4′), 40.8 (C-8′), 119.2 (C-8), 122.3 (C-3), 122.6 (C-2′), 125.0 (C-6), 125.3 (C-6′), 125.4 (C-10′), 125.6 (C-4a), 126.2 (C-5), 132.0 (C-11′), 133.0 (C-7), 136.1 (C-7′), 138.4 (C-3′), 138.9 (C-2), 141.0 (C-8a), 179.0 (C-4). HR-ESIMS: *m/z* 350.2438 [M+H]^+^ (calcd. for C_24_H_32_NO 350.2406; Δppm 9.1). Analytical data in agreement with those previously reported [16].

6-Methyl aurachin D (**3**). ^1^H NMR (700 MHz, methanol-*d*_4_), δH [ppm] (*J* [Hz]) 1.50 (3 H, d, *J* 1.0, H-15′), 1.55 (3 H, d, *J* 1.3, H-14′), 1.61 (3 H, d, *J* 1.3, H-12′), 1.80 (3 H, d, *J* 1.3, H-13′), 1.88 (2 H, m, H-8′), 1.95 (2 H, m, H-9′), 2.02 (2 H, m, H-4′), 2.10 (2 H, m, H-5′), 2.45 (3 H, s, H-9), 2.46 (3 H, s, H-10), 3.40 (2 H, d, *J* 6.8, H-1′), 5.00 (1 H, m, H-10′), 5.05 (1 H, m, H-6′), 5.10 (1 H, m, H-2′), 7.42 (1 H, d, *J* 8.4, H-8), 7.48 (1 H, dd, *J* 2.2, 8.4, H-7), 8.02 (1 H, d, *J* 2.2, H-5). ^13^C NMR (175 MHz, methanol-*d*_4_), δC [ppm] 16.1 (C-14′), 16.3 (C-13′), 17.6 (C-15′), 18.2 (C-9), 21.3 (C-10), 24.8 (C-1′), 25.8 (C-12′), 27.3 (C-5′), 27.8 (C-9′), 40.7 (C-4′), 40.8 (C-8′), 118.6 (C-8), 120.6 (C-3), 124.0 (C-2′), 125.0 (C-4a), 125.3 (C-6′), 125.4 (C-10′), 125.4 (C-5), 132.0 (C-11′), 134.2 (C-7), 134.6 (C-6), 135.8 (C-3′), 135.9 (C-7′), 138.7 (C-8a), 149.4 (C-2), 178.2 (C-4). HR-ESIMS: *m/z* 378.2739 [M+H]^+^ (calcd. for C_26_H_36_NO 378.2719; Δppm 5.3). Analytical data in agreement with those previously reported [16].

7-Methyl aurachin D (**4**). ^1^H NMR (600 MHz, methanol-*d*_4_), δH [ppm] (*J* [Hz]) 1.50 (3 H, d, *J* 0.9, H-15′), 1.55 (3 H, d, *J* 1.1, H-14′), 1.61 (3 H, d, *J* 1.3, H-12′), 1.80 (3 H, d, *J* 1.2, H-13′), 1.88 (2 H, m, H-8′), 1.94 (2 H, m, H-9′), 2.01 (2 H, m, H-4′), 2.10 (2 H, m, H-5′), 2.45 (3 H, s, H-9), 2.47 (3 H, s, H-10), 3.39 (2 H, d, *J* 6.9, H-1′), 5.00 (1 H, m, H-10′), 5.05 (1 H, m, H-6′), 5.09 (1 H, m, H-2′), 7.19 (1 H, dd, *J* 1.7, 8.4, H-6), 7.29 (1 H, d, *J* 1.7, H-8), 8.11 (1 H, d, *J* 8.4, H-5). ^13^C NMR (150 MHz, methanol-*d*_4_), δC [ppm] 16.1 (C-14′), 16.3 (C-13′), 17.6 (C-15′), 18.2 (C-9), 21.8 (C-10), 24.7 (C-1′), 25.8 (C-12′), 27.3 (C-5′), 27.8 (C-9′), 40.7 (C-4′), 40.8 (C-8′), 118.0 (C-8), 120.5 (C-3), 123.1 (C-4a), 123.9 (C-2′), 125.3 (C-6′), 125.4 (C-10′), 126.3 (C-5), 126.4 (C-6), 132.0 (C-11′), 135.8 (C-3′), 135.9 (C-7′), 140.8 (C-8a), 143.6 (C-7), 149.4 (C-2), 178.4 (C-4). HR-ESIMS: *m/z* 378.2729 [M+H]^+^ (calcd. for C_26_H_36_NO 378.2719; Δppm 2.6).

6-Methoxy aurachin D (**5**). ^1^H NMR (600 MHz, methanol-*d*_4_), δH [ppm] (*J* [Hz]) 1.50 (3 H, d, *J* 0.7, H-15′), 1.56 (3 H, d, *J* 1.0, H-14′), 1.61 (3 H, d, *J* 1.3, H-12′), 1.81 (3 H, d, *J* 1.2, H-13′), 1.88 (2 H, m, H-8′), 1.94 (2 H, m, H-9′), 2.03 (2 H, m, H-4′), 2.10 (2 H, m, H-5′), 2.46 (3 H, s, H-9), 3.42 (2 H, d, *J* 6.7, H-1′), 3.90 (3 H, s, H-10), 5.00 (1 H, m, H-10′), 5.05 (1 H, m, H-6′), 5.11 (1 H, m, H-2′), 7.27 (1 H, dd, *J* 2.9, 9.0, H-7), 7.46 (1 H, d, *J* 9.0, H-8), 7.64 (1 H, d, *J* 2.9, H-5). ^13^C NMR (150 MHz, methanol-*d*_4_), δC [ppm] 16.1 (C-14′), 16.3 (C-13′), 17.6 (C-15′), 18.2 (C-9), 24.9 (C-1′), 25.8 (C-12′), 27.3 (C-5′), 27.8 (C-9′), 40.7 (C-4′), 40.8 (C-8′), 56.0 (C-10), 104.9 (C-5), 120.1 (C-3), 120.4 (C-8), 123.8 (C-7), 123.9 (C-2′), 125.3 (C-6′), 125.4 (C-10′), 126.0 (C-4a), 132.0 (C-11′), 135.4 (C-8a), 135.8 (C-3′), 135.9 (C-7′), 148.8 (C-2), 157.8 (C-6), 177.6 (C-4). HR-ESIMS: *m/z* 394.2691 [M+H]^+^ (calcd. for C_26_H_36_NO_2_ 394.2668; Δppm 5.8). Analytical data in agreement with those previously reported [12,20].

7-Methoxy aurachin D (**6**). ^1^H NMR (600 MHz, methanol-*d*_4_), δH [ppm] (*J* [Hz]) 1.50 (3 H, d, *J* 1.0, H-15′), 1.56 (3 H, d, *J* 1.2, H-14′), 1.61 (3 H, d, *J* 1.3, H-12′), 1.81 (3 H, d, *J* 1.2, H-13′), 1.88 (2 H, m, H-8′), 1.94 (2 H, m, H-9′), 2.03 (2 H, m, H-4′), 2.11 (2 H, m, H-5′), 2.52 (3 H, s, H-9), 3.43 (2 H, d, *J* 7.2, H-1′), 3.93 (3 H, s, H-10), 4.99 (1 H, m, H-10′), 5.05 (1 H, m, H-6′), 5.09 (1 H, m, H-2′), 6.98 (1 H, d, *J* 2.4, H-8), 7.07 (1 H, dd, *J* 2.4, 9.1, H-6), 8.17 (1 H, d, *J* 9.1, H-5). ^13^C NMR (150 MHz, methanol-*d*_4_), δC [ppm] 16.1 (C-14′), 16.4 (C-13′), 17.6 (C-15′), 18.4 (C-9), 24.7 (C-1′), 25.8 (C-12′), 27.3 (C-5′), 27.8 (C-9′), 40.7 (C-8′), 40.8 (C-4′), 56.3 (C-10), 99.1 (C-8), 116.9 (C-6), 119.9 (C-3), 123.1 (C-2′), 125.3 (C-10′), 125.3 (C-6′), n.d. (C-4a), 127.5 (C-5), 132.0 (C-11′), 136.0 (C-7′), 136.6 (C-3′), 142.3 (C-8a), 151.2 (C-2), 164.5 (C-7), 175.1 (C-4). HR-ESIMS: *m/z* 394.2690 [M+H]^+^ (calcd. for C_26_H_36_NO_2_ 394.2668; Δppm 5.6). Analytical data in agreement with those previously reported [20].

6-Fluoro aurachin D (**7**). ^1^H NMR (700 MHz, methanol-*d*_4_), δH [ppm] (*J* [Hz]) 1.51 ppm (3H, d, *J*_H,H_ 1.5, H-15′), 1.55 (3H, d, *J*_H,H_ 1.3, H-14′), 1.61 (3H, d, *J*_H,H_ 1.4, H-12′), 1.81 (3H, d, *J*_H,H_ 1.3, H-13′), 1.88 (2H, m, H-8′), 1.94 (2H, m, H-9′), 2.02 (2H, m, H-4′), 2.09 (2H, m, H-5′), 2.47 (3H, s, H-9), 3.40 (2H, d, *J*_H,H_ 6.9, H-1′), 4.99 (1H, ddt, *J*_H,H_ 1.4, 1.5, 7.0, H-10′), 5.05 (1H, dt, *J*_H,H_ 1.3, 6..9, H-6′), 5.09 (1H, dt, *J*_H,H_ 1.3, 6.9, H-2′), 7.44 (1H, ddd, *J*_H,H_ 2.9, 9.1, *J*_H,F_ 8.1, H-7), 7.56 (1H, dd, *J*_H,H_ 9.1, *J*_H,F_ 4.5, H-8), 7.84 (1H, dd, *J*_H,H_ 2.9, *J*_H,F_ 9.5, H-5). ^13^C NMR (175 MHz, methanol-*d*_4_), δC [ppm] 16.1 (C-14′), 16.3 (C-13′), 17.6 (C-15′), 18.3 (C-9), 24.8 (C-1′), 25.8 (C-12′), 27.3 (C-5′), 27.8 (C-9′), 40.7 (C-8′), 40.8 (C-4′), 110.2 (d, *J*_C,F_ 23.1, C-5), 120.4 (C-3), 121.3 (d, *J*_C,F_ 8.5, C-8), 121.5 (d, *J*_C,F_ 26.7, C-7), 123.6 (C-2′), 125.3 (C-10′), 125.4 (C-6′), 126.1 (d, *J*_C,F_ 7.1, C-4a), 132.1 (C-11′), 135.9 (C-7′), 136.1 (C-3′), 137.2 (C-8a), 149.9 (C-2), 160.5 (d, *J*_C,F_ 241.7, C-6), 177.6 (C-4). HR-ESIMS: *m/z* 382.2485 [M+H]^+^ (calcd. for C_25_H_33_FNO 382.2468; Δppm 4.4). Analytical data in agreement with those previously reported [14,20].

6-Chloro aurachin D (**8**). ^1^H NMR (600 MHz, methanol-*d*_4_), δH [ppm] (*J* [Hz]) 1.50 (3 H, d, *J* 0.9, H-15′), 1.55 (3 H, d, *J* 1.0, H-14′), 1.61 (3 H, d, *J* 1.3, H-12′), 1.80 (3 H, d, *J* 1.1, H-13′), 1.87 (2 H, m, H-8′), 1.93 (2 H, m, H-9′), 2.02 (2 H, m, H-4′), 2.10 (2 H, m, H-5′), 2.46 (3 H, s, H-9), 3.39 (2 H, d, *J* 7.0, H-1′), 4.99 (1 H, m, H-10′), 5.04 (1 H, m, H-6′), 5.08 (1 H, m, H-2′), 7.50 (1 H, d, *J* 8.9, H-8), 7.60 (1 H, dd, *J* 2.5, 8.9, H-7), 8.18 (1 H, d, *J* 2.5, H-5). ^13^C NMR (150 MHz, methanol-*d*_4_), δC [ppm] 16.2 (C-14′), 16.3 (C-13′), 17.6 (C-15′), 18.3 (C-9), 24.8 (C-1′), 25.8 (C-12′), 27.3 (C-5′), 27.8 (C-9′), 40.7 (C-4′), 40.8 (C-8′), 120.8 (C-8), 121.3 (C-3), 123.5 (C-2′), 125.3 (C-6′), 125.3 (C-10′), 125.5 (C-5), 125.9 (C-4a), 130.3 (C-6), 132.0 (C-11′), 132.9 (C-7), 135.9 (C-7′), 136.1 (C-3′), 139.0 (C-8a), 150.1 (C-2), 177.3 (C-4). HR-ESIMS: *m/z* 398.2188 [M+H]^+^ (calcd. for C_25_H_33_ClNO 398.2172; Δppm 4.0). Analytical data in agreement with those previously reported [20].

7-Chloro aurachin D (**9**). ^1^H NMR (600 MHz, methanol-*d*_4_), δH [ppm] (*J* [Hz]) 1.49 (3 H, d, *J* 0.8, H-15′), 1.55 (3 H, d, *J* 1.0, H-14′), 1.61 (3 H, d, *J* 1.3, H-12′), 1.80 (3 H, d, *J* 1.1, H-13′), 1.87 (2 H, m, H-8′), 1.92 (2 H, m, H-9′), 2.02 (2 H, m, H-4′), 2.10 (2 H, m, H-5′), 2.45 (3 H, s, H-9), 3.37 (2 H, d, *J* 6.8, H-1′), 4.98 (1 H, m, H-10′), 5.04 (1 H, m, H-6′), 5.08 (1 H, m, H-2′), 7.31 (1 H, dd, *J* 2.0, 8.8, H-6), 7.51 (1 H, d, *J* 2.0, H-8), 8.18 (1 H, d, *J* 8.8, H-5). ^13^C NMR (150 MHz, methanol-*d*_4_), δC [ppm] 16.2 (C-14′), 16.3 (C-13′), 17.6 (C-15′), 18.3 (C-9), 24.7 (C-1′), 25.8 (C-12′), 27.2 (C-5′), 27.8 (C-9′), 40.7 (C-4′), 40.8 (C-8′), 118.0 (C-8), 121.4 (C-3), 123.5 (C-2′), 123.6 (C-4a), 125.0 (C-6), 125.3 (C-6′), 125.3 (C-10′), 128.5 (C-5), 132.0 (C-11′), 135.9 (C-7′), 136.0 (C-3′), 138.6 (C-7), 141.2 (C-8a), 150.1 (C-2), 178.0 (C-4). HR-ESIMS: *m/z* 398.2191 [M+H]^+^ (calcd. for C_25_H_33_ClNO 398.2172; Δppm 4.8). Analytical data in agreement with those previously reported [16,20].

6-Bromo aurachin D (**10**). ^1^H NMR (600 MHz, methanol-*d*_4_), δH [ppm] (*J* [Hz]) 1.50 (3 H, d, *J* 0.8, H-15′), 1.55 (3 H, d, *J* 1.0, H-14′), 1.61 (3 H, d, *J* 1.3, H-12′), 1.80 (3 H, d, *J* 1.1, H-13′), 1.87 (2 H, m, H-8′), 1.93 (2 H, m, H-9′), 2.02 (2 H, m, H-4′), 2.10 (2 H, m, H-5′), 2.46 (3 H, s, H-9), 3.38 (2 H, d, *J* 6.9, H-1′), 4.99 (1 H, m, H-10′), 5.04 (1 H, m, H-6′), 5.08 (1 H, m, H-2′), 7.44 (1 H, d, *J* 8.9, H-8), 7.72 (1 H, dd, *J* 2.2, 8.9, H-7), 8.34 (1 H, d, *J* 2.2, H-5). ^13^C NMR (150 MHz, methanol-*d*_4_), δC [ppm] 16.2 (C-14′), 16.3 (C-13′), 17.6 (C-15′), 18.3 (C-9), 24.8 (C-1′), 25.8 (C-12′), 27.3 (C-5′), 27.8 (C-9′), 40.7 (C-4′), 40.8 (C-8′), 117.7 (C-4a), 120.9 (C-8), 121.4 (C-3), 123.5 (C-2′), 125.3 (C-6′), 125.3 (C-10′), 126.4 (C-6), 128.8 (C-5), 132.0 (C-11′), 135.5 (C-7), 135.9 (C-7′), 136.1 (C-3′), 139.3 (C-8a), 150.1 (C-2), 177.2 (C-4). HR-ESIMS: *m/z* 442.1668 [M+H]^+^ (calcd. for C_25_H_33_BrNO 442.1667; Δppm 0.2).

### 4.6. Biological Tests

#### 4.6.1. Antiplasmodial Assay

Antiplasmodial activity was determined against the NF54 strain of *Plasmodium falciparum* using a modified ^3^H-hypoxanthine incorporation assay. Briefly, infected erythrocytes were exposed to serial drug dilutions in microtiter plates for 48 h at 37 °C in a gas mixture with reduced oxygen and elevated carbon dioxide concentrations [38,39]. ^3^H hypoxanthine was added to each well, and after further incubation for 24 h, the wells were harvested on glass fiber filters and counted in a liquid scintillation counter. From the sigmoidal inhibition curve, the IC_50_ value was calculated. Chloroquine was used as positive control in each test series.

#### 4.6.2. Antitrypanosomal Assays

Activity against *Trypanosoma brucei rhodesiense* (strain STIB 900) was evaluated according to a previously established protocol [39,40]. Parasites were grown axenically in culture medium supplemented with horse serum. Following a 3-day exposure to test compounds, the viability of tryptomastigote parasites was quantified using the dye resazurin by monitoring the reductive environment of living cells. Fluorescence development was expressed as percentage of the control, and IC_50_ values were calculated. Melarsoprol was included as positive control.

Activity against *Trypanosoma cruzi* was evaluated according to a procedure described by Buckner and coworkers [41]. The strain Tulahuen C4 of *T. cruzi*, which had been transfected with the *β*-galactosidase *lacZ* gene, was cultivated for 4 days on rat skeletal myoblasts (5% CO_2_, 37 °C) in the presence of drug. For measurement of the IC_50_, the substrate chlorophenol red-*β*-d-galactopyranoside was added. The color reaction that developed during the following 2–4 h was quantified photometrically employing an ELISA reader. Benznidazole was included in each test series as positive control.

#### 4.6.3. Antileishmanial Assay

Evaluation of antileishmanial activity was carried out with axenically grown amastigotes of *Leishmania donovani* MHOM-ET-67/L82, as previously described [39]. For this, the amastigotes were cultured at 37 °C and pH 5.4 in SM medium [42] supplemented with 10% heat-inactivated fetal bovine serum under an atmosphere of 5% CO_2_ in air. Following a 3-day exposure to test compounds, the viability of amastigote parasites was quantified using the dye resazurin by monitoring the reductive environment of living cells. Fluorescence development was expressed as percentage of the control, and IC_50_ values were calculated. Miltefosine was used as positive control.

#### 4.6.4. Cytotoxicity Assay

Cytotoxicity was tested with L6 cells (ATCC CRL-1458), a primary cell line derived from rat skeletal myoblasts, as previously described [38]. For this, L6 cells were grown in culture medium supplemented with heat-inactivated fetal bovine serum under an atmosphere of 5% CO_2_ in air. Following a 3-day exposure to test compounds, the viability of L6 cells was quantified using the dye resazurin by monitoring the reductive environment of living cells. Fluorescence development was expressed as percentage of the control, and IC_50_ values were calculated. Podophyllotoxin was used as reference.

## Figures and Tables

**Scheme 1:**
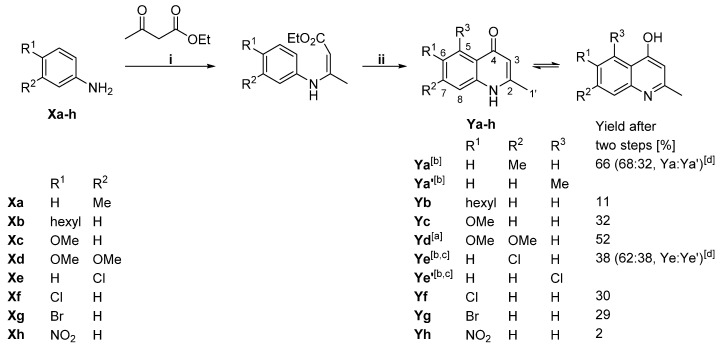
Reaction conditions: (i) Zn(ClO_4_)_2_·6H_2_O, MgSO_4_, EtOH, rt, 12–48 h. (ii) Ph_2_O, 4Å MS, 250 °C, 1 h. ^[a]^ Reaction conditions: (i) CAN, EtOH, rt, 36 h. (ii) Ph_2_O, 190 °C, 280 W, 45 min. ^[b]^ Obtained as a mixture of regioisomers. ^[c]^ Separated via preparative HPLC. ^[d]^ Ratio of regioisomers determined via ^1^H NMR (Figures S1 and S10).

**Table 1:** Whole-cell biotransformation of 1*H*-quinolin-4-one substrates into aurachin D analogs. The relative activity was defined as the ratio of the conversion rate of a substrate to that of 2-methyl-1*H*-quinolin-4-one.

Functional Groups on 1*H*-Quinolin-4-One Substrate	Product	Molar Yield Coefficient Y_P/S_ [%]	Relative Activity [%]
2-methyl	aurachin D (**1**)	11	100.0
-	2-desmethyl aurachin D (**2**)	27	245.5
2,6-dimethyl	6-methyl aurachin D (**3**)	5	45.5
2,7-dimethyl (**Ya**)	7-methyl aurachin D (**4**)	14	127.3
2-methyl, 6-hexyl (**Yb**)	-	0	0
2-methyl, 6-methoxy (**Yc**)	6-methoxy aurachin D (**5**)	2	18.2
2-methyl, 7-methoxy	7-methoxy aurachin D (**6**)	1	9.1
2-methyl, 6,7-dimethoxy (**Yd**)	-	0	0
2-methyl, 6-fluoro	6-fluoro aurachin D (**7**)	13	118.2
2-methyl, 6-chloro (**Yf**)	6-chloro aurachin D (**8**)	8	72.7
2-methyl, 7-chloro (**Ye**)	7-chloro aurachin D (**9**)	19	172.7
2-methyl, 6-bromo (**Yg**)	6-bromo aurachin D (**10**)	9	81.8
2-methyl, 6-nitro (**Yh**)	-	0	0

**Table 2:** Activities of aurachin D and its derivatives against parasitic protozoa and mammalian cells. IC_50_ values are given in μM. ^a^ The antileishmanial selectivity index (S.I.) was determined as IC_50_ (L6)/IC_50_ (*L. donovani*).

Test or Reference Compound	IC_50_ *P. falciparum* NF54	IC_50_ *T. brucei rhodesiense* STIB 900	IC_50_ *T. cruzi* Tulahuen C4	IC_50_ *L. donovani* MHOM-ET-67/L82	IC_50_ Rat Myoblast L6 Cells	Antileishmanial S.I.
**1**	0.012	4.5	1.3	0.044	130.7	2969.5
**2**	0.006	9.5	2.4	1.5	20.4	13.2
**3**	0.514	43.2	7.6	4.3	18.6	4.3
**4**	0.007	163.8	45.7	9.1	65.3	7.2
**5**	0.866	40.3	19.3	7.3	118.3	16.2
**6**	0.089	44.8	21.9	17.1	80.8	4.7
**7**	0.061	37.2	1.8	0.617	125.8	203.9
**8**	0.021	37.4	11.2	6.2	117.5	18.9
**9**	0.070	104.9	41.8	9.2	54.7	5.9
**10**	0.119	40.2	22.6	13.9	107.7	7.7
Chloroquine	0.008					
Melarsoprol		0.013				
Benznidazole			2.5			
Miltefosine				0.732		
Podophyllotoxin					0.022	

^a^ Values represent the average of four determinations (two determinations of two independent experiments).

## Data Availability

The data presented in this study are available in the Supplementary Materials).

## Supplementary Materials

The following supporting information can be downloaded at: https://www.mdpi.com/article/10.3390/molecules28031066/s1, Figure S1: ^1^H NMR spectrum (600 MHz, methanol-*d*_4_) of a mixture of **Ya** and **Ya’**; Figure S2: ^1^H-decoupled ^13^C NMR spectrum (150 MHz, methanol-*d*_4_) of a mixture of **Ya** and **Ya’**; Figure S3: ^1^H NMR spectrum (600 MHz, methanol-*d*_4_) of **Ya**; Figure S4: ^1^H NMR spectrum (600 MHz, methanol-*d*_4_) of **Yb**; Figure S5: ^1^H-decoupled ^13^C NMR spectrum (150 MHz, methanol-*d*_4_) of **Yb**; Figure S6: ^1^H NMR spectrum (600 MHz, methanol-*d*_4_) of **Yc**; Figure S7: ^1^H-decoupled ^13^C NMR spectrum (150 MHz, methanol-*d*_4_) of **Yc**; Figure S8: ^1^H NMR spectrum (600 MHz, methanol-*d*_4_) of **Yd**; Figure S9: ^1^H-decoupled ^13^C NMR spectrum (150 MHz, methanol-*d*_4_) of **Yd**; Figure S10: ^1^H NMR spectrum (600 MHz, methanol-*d*_4_) of a mixture of **Ye** and **Ye’**; Figure S11: ^1^H-decoupled ^13^C NMR spectrum (150 MHz, methanol-*d*_4_) of a mixture of **Ye** and **Ye’**; Figure S12: ^1^H NMR spectrum (600 MHz, methanol-*d*_4_) of **Ye**; Figure S13: ^1^H NMR spectrum (600 MHz, methanol-*d*_4_) of **Ye’**; Figure S14: ^1^H NMR spectrum (600 MHz, methanol-*d*_4_) of **Yg**; Figure S15: ^1^H-decoupled ^13^C NMR spectrum (150 MHz, methanol-*d*_4_) of **Yg**; Figure S16: ^1^H NMR spectrum (600 MHz, DMSO-*d*_6_) of **Yh**; Figure S17: ^1^H-decoupled ^13^C NMR spectrum (150 MHz, DMSO-*d*_6_) of **Yh**; Figure S18: ^1^H NMR spectrum (700 MHz, methanol-*d*_4_) of **1**; Figure S19: ^1^H-decoupled ^13^C NMR spectrum (175 MHz, methanol-*d*_4_) of **1**; Figure S20: ^1^H NMR spectrum (700 MHz, methanol-*d*_4_) of **2**; Figure S21: ^1^H-decoupled ^13^C NMR spectrum (175 MHz, methanol-*d*_4_) of **2**; Figure S22: ^1^H NMR spectrum (700 MHz, methanol-*d*_4_) of **3**; Figure S23: ^1^H-decoupled ^13^C NMR spectrum (175 MHz, methanol-*d*_4_) of **3**; Figure S24: ^1^H NMR spectrum (600 MHz, methanol-*d*_4_) of **4**; Figure S25: ^1^H-decoupled ^13^C NMR spectrum (150 MHz, methanol-*d*_4_) of **4**; Figure S26: COSY spectrum (methanol-*d*_4_) of **4**; Figure S27: HSQC spectrum (methanol-*d*_4_) of **4**; Figure S28: HMBC spectrum (methanol-*d*_4_) of **4**; Figure S29: ^1^H NMR spectrum (600 MHz, methanol-*d*_4_) of **5**; Figure S30: ^1^H-decoupled ^13^C NMR spectrum (150 MHz, methanol-*d*_4_) of **5**; Figure S31: ^1^H NMR spectrum (600 MHz, methanol-*d*_4_) of **6**; Figure S32: ^1^H-decoupled ^13^C NMR spectrum (150 MHz, methanol-*d*_4_) of **6**; Figure S33: ^1^H NMR spectrum (700 MHz, methanol-*d*_4_) of **7**; Figure S34: ^1^H-decoupled ^13^C NMR spectrum (175 MHz, methanol-*d*_4_) of **7**; Figure S35: ^1^H NMR spectrum (600 MHz, methanol-*d*_4_) of **8**; Figure S36: ^1^H-decoupled ^13^C NMR spectrum (150 MHz, methanol-*d*_4_) of **8**; Figure S37: ^1^H NMR spectrum (600 MHz, methanol-*d*_4_) of **9**; Figure S38: ^1^H-decoupled ^13^C NMR spectrum (150 MHz, methanol-*d*_4_) of **9**; Figure S39: ^1^H NMR spectrum (600 MHz, methanol-*d*_4_) of **10**; Figure S40: ^1^H-decoupled ^13^C NMR spectrum (150 MHz, methanol-*d*_4_) of **10**; Figure S41: COSY spectrum (methanol-*d*_4_) of **10**; Figure S42: HSQC spectrum (methanol-*d*_4_) of **10**; Figure S43: HMBC spectrum (methanol-*d*_4_) of **10**.

## Sample Availability

The recombinant *E. coli* strain used in this study is available upon request.
