# Supplementary material for: Generation of Aurachin Derivatives by Whole-Cell Biotransformation and Evaluation of Their Antiprotozoal Properties
Source: Molecules. 2023 Jan 20;28(3):1066. doi: 10.3390/molecules28031066 (PMC9919615; doi:10.3390/molecules28031066)
Supplement: Supplementary file 1 [file molecules-28-01066-s001.zip › molecules-2149751-supplementary.pdf]

Generation of Aurachin Derivatives by Whole-Cell Biotransformation and Evaluation of their Antiprotozoal Properties

Sebastian Kruth [1], Cindy J.-M. Zimmermann [2], Katharina Kuhr [1], Wolf Hiller [3], Stephan Lütz [4], Jörg Pietruszka [2,5], Marcel Kaiser [6,7] and Markus Nett [1,*]

1 Department of Biochemical and Chemical Engineering, Laboratory of Technical Biology, TU Dortmund University, 44227 Dortmund, Germany

2 Institute of Bioorganic Chemistry, Heinrich-Heine-University Düsseldorf at Forschungszentrum Jülich, and Bioeconomy Science Center (BioSC), 52426 Jülich, Germany

3 Department of Chemistry and Chemical Biology, NMR Laboratory, TU Dortmund University, 44227 Dortmund, Germany

4 Department of Biochemical and Chemical Engineering, Laboratory of Bioprocess Engineering, TU Dortmund University, 44227 Dortmund, Germany

5 Institute of Bio- and Geosciences: Biotechnology (IBG-1), Forschungszentrum Jülich, 52428 Jülich, Germany

6 Swiss Tropical and Public Health Institute, 4123 Allschwil, Switzerland

7 Swiss Tropical and Public Health Institute, University of Basel, Petersplatz 1, 4002 Basel, Switzerland

Table of contents

Figure S1. ^{1}H NMR spectrum (600 MHz, methanol-d_4) of a mixture of **Ya** and **Ya′** 3

Figure S2. ^{1}H-decoupled ^{13}C NMR spectrum (150 MHz, methanol-d_4) of a mixture of **Ya** and **Ya′** 3

Figure S3. ^{1}H NMR spectrum (600 MHz, methanol-d_4) of **Ya** 4

Figure S4. ^{1}H NMR spectrum (600 MHz, methanol-d_4) of **Yb** 5

Figure S5. ^{1}H-decoupled ^{13}C NMR spectrum (150 MHz, methanol-d_4) of **Yb** 5

Figure S6. ^{1}H NMR spectrum (600 MHz, methanol-d_4) of **Yc** 6

Figure S7. ^{1}H-decoupled ^{13}C NMR spectrum (150 MHz, methanol-d_4) of **Yc** 6

Figure S8. ^{1}H NMR spectrum (600 MHz, methanol-d_4) of **Yd** 7

Figure S9. ^{1}H-decoupled ^{13}C NMR spectrum (150 MHz, methanol-d_4) of **Yd** 7

Figure S10. ^{1}H NMR spectrum (600 MHz, methanol-d_4) of a mixture of **Ye** and **Ye′** 8

Figure S11. ^{1}H-decoupled ^{13}C NMR spectrum (150 MHz, methanol-d_4) of a mixture of **Ye** and **Ye′** 8

Figure S12. ^{1}H NMR spectrum (600 MHz, methanol-d_4) of **Ye** 9

Figure S13. ^{1}H NMR spectrum (600 MHz, methanol-d_4) of **Ye′** 9

Figure S14. ^{1}H NMR spectrum (600 MHz, methanol-d_4) of **Yg** 10

Figure S15. ^{1}H-decoupled ^{13}C NMR spectrum (150 MHz, methanol-d_4) of **Yg** 10
Figure S16. ^{1}H NMR spectrum (600 MHz, DMSO-d_6) of **Yh** 11
Figure S17. ^{1}H-decoupled ^{13}C NMR spectrum (150 MHz, DMSO-d_6) of **Yh** 11
Figure S18. ^{1}H NMR spectrum (700 MHz, methanol-d_4) of **1** 12
Figure S19. ^{1}H-decoupled ^{13}C NMR spectrum (175 MHz, methanol-d_4) of **1** 12
Figure S20. ^{1}H NMR spectrum (700 MHz, methanol-d_4) of **2** 13
Figure S21. ^{1}H-decoupled ^{13}C NMR spectrum (175 MHz, methanol-d_4) of **2** 13
Figure S22. ^{1}H NMR spectrum (700 MHz, methanol-d_4) of **3** 14
Figure S23. ^{1}H-decoupled ^{13}C NMR spectrum (175 MHz, methanol-d_4) of **3** 14
Figure S24. ^{1}H NMR spectrum (600 MHz, methanol-d_4) of **4** 15
Figure S25. ^{1}H-decoupled ^{13}C NMR spectrum (150 MHz, methanol-d_4) of **4** 15
Figure S26. COSY spectrum (methanol-d_4) of **4** 16
Figure S27. HSQC spectrum (methanol-d_4) of **4** 16
Figure S28. HMBC spectrum (methanol-d_4) of **4** 17
Figure S29. ^{1}H NMR spectrum (600 MHz, methanol-d_4) of **5** 18
Figure S30. ^{1}H-decoupled ^{13}C NMR spectrum (150 MHz, methanol-d_4) of **5** 18
Figure S31. ^{1}H NMR spectrum (600 MHz, methanol-d_4) of **6** 19
Figure S32. ^{1}H-decoupled ^{13}C NMR spectrum (150 MHz, methanol-d_4) of **6** 19
Figure S33. ^{1}H NMR spectrum (700 MHz, methanol-d_4) of **7** 20
Figure S34. ^{1}H-decoupled ^{13}C NMR spectrum (175 MHz, methanol-d_4) of **7** 20
Figure S35. ^{1}H NMR spectrum (600 MHz, methanol-d_4) of **8** 21
Figure S36. ^{1}H-decoupled ^{13}C NMR spectrum (150 MHz, methanol-d_4) of **8** 21
Figure S37. ^{1}H NMR spectrum (600 MHz, methanol-d_4) of **9** 22
Figure S38. ^{1}H-decoupled ^{13}C NMR spectrum (150 MHz, methanol-d_4) of **9** 22
Figure S39. ^{1}H NMR spectrum (600 MHz, methanol-d_4) of **10** 23
Figure S40. ^{1}H-decoupled ^{13}C NMR spectrum (150 MHz, methanol-d_4) of **10** 23
Figure S41. COSY spectrum (methanol-d_4) of **10** 24
Figure S42. HSQC spectrum (methanol-d_4) of **10** 24
Figure S43. HMBC spectrum (methanol-d_4) of **10** 25

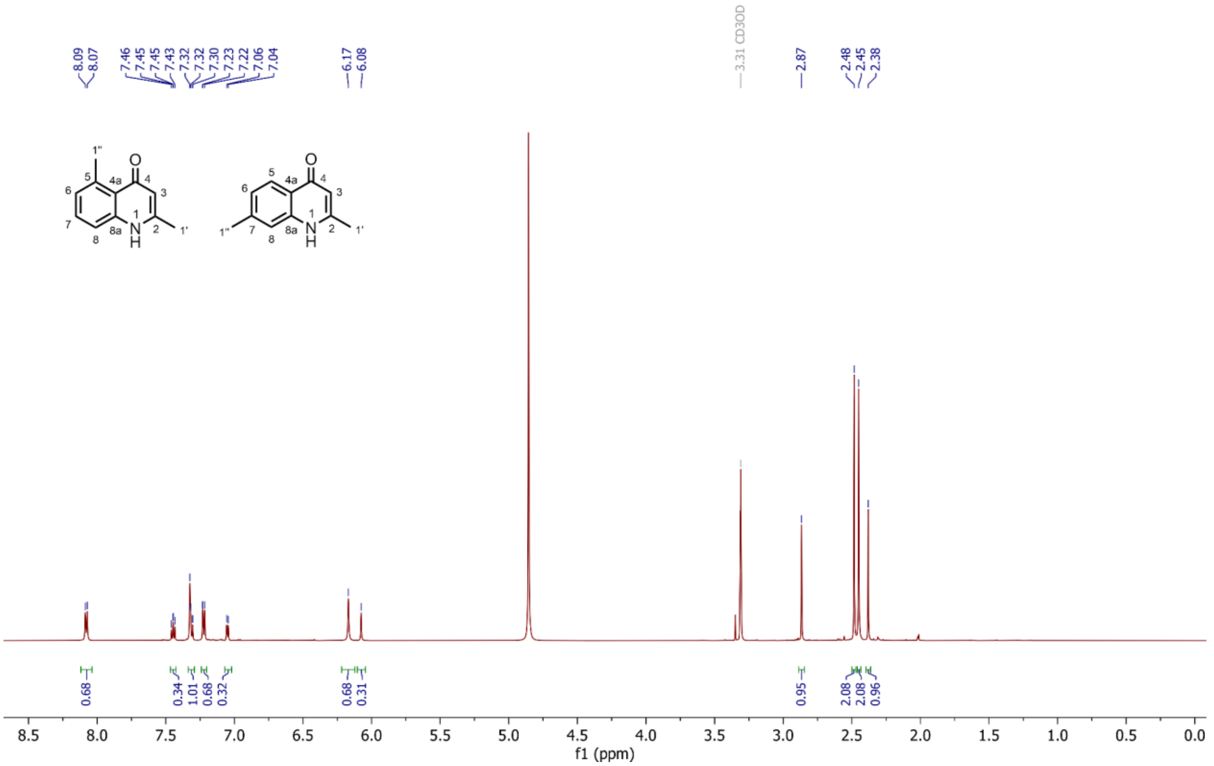

Figure S1. ^{1}H NMR spectrum (600 MHz, methanol-d_4) of a mixture of 2,7-dimethyl-1*H*-quinolin-4-one (**Ya**) and 2,5-dimethyl-1*H*-quinolin-4-one (**Ya'**).

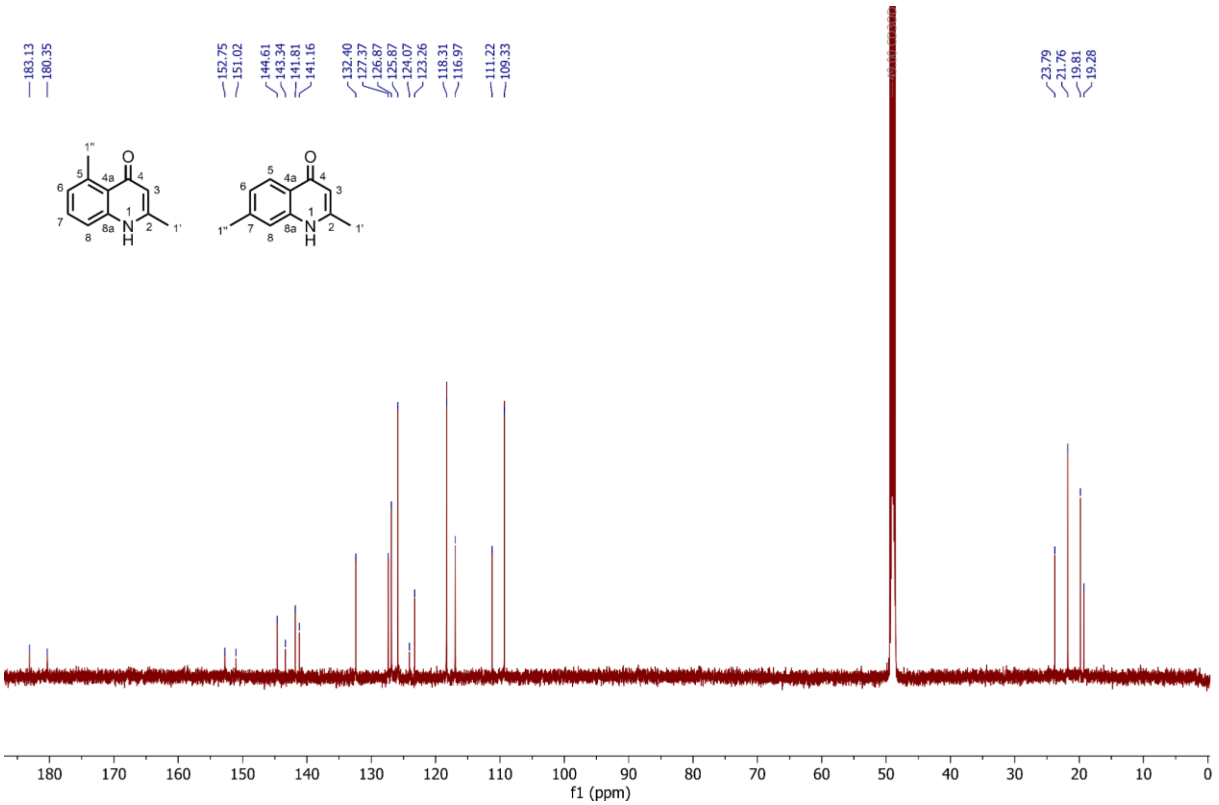

Figure S2. ^{1}H-decoupled ^{13}C NMR spectrum (150 MHz, methanol-d_4) of a mixture of 2,7-dimethyl-1*H*-quinolin-4-one (**Ya**) and 2,5-dimethyl-1*H*-quinolin-4-one (**Ya'**).

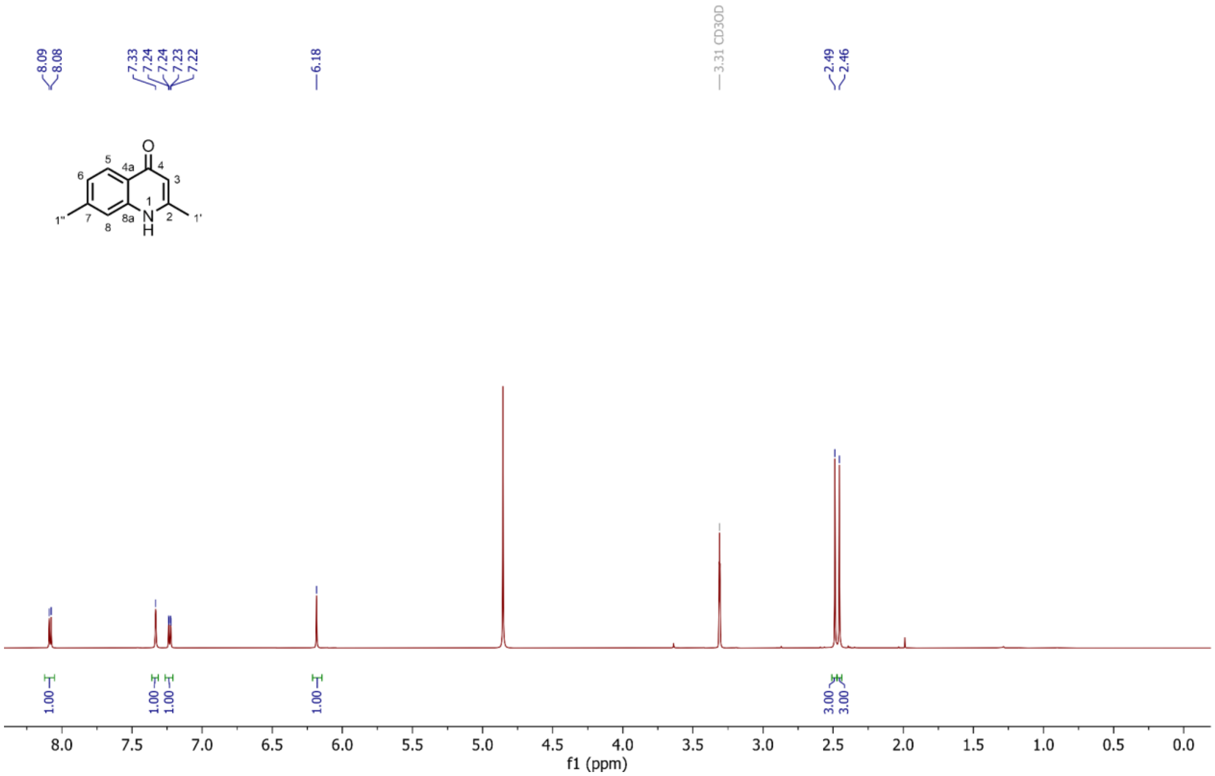

Figure S3. ^{1}H NMR spectrum (600 MHz, methanol-d_4) of 2,7-dimethyl-1*H*-quinolin-4-one (**Ya**).

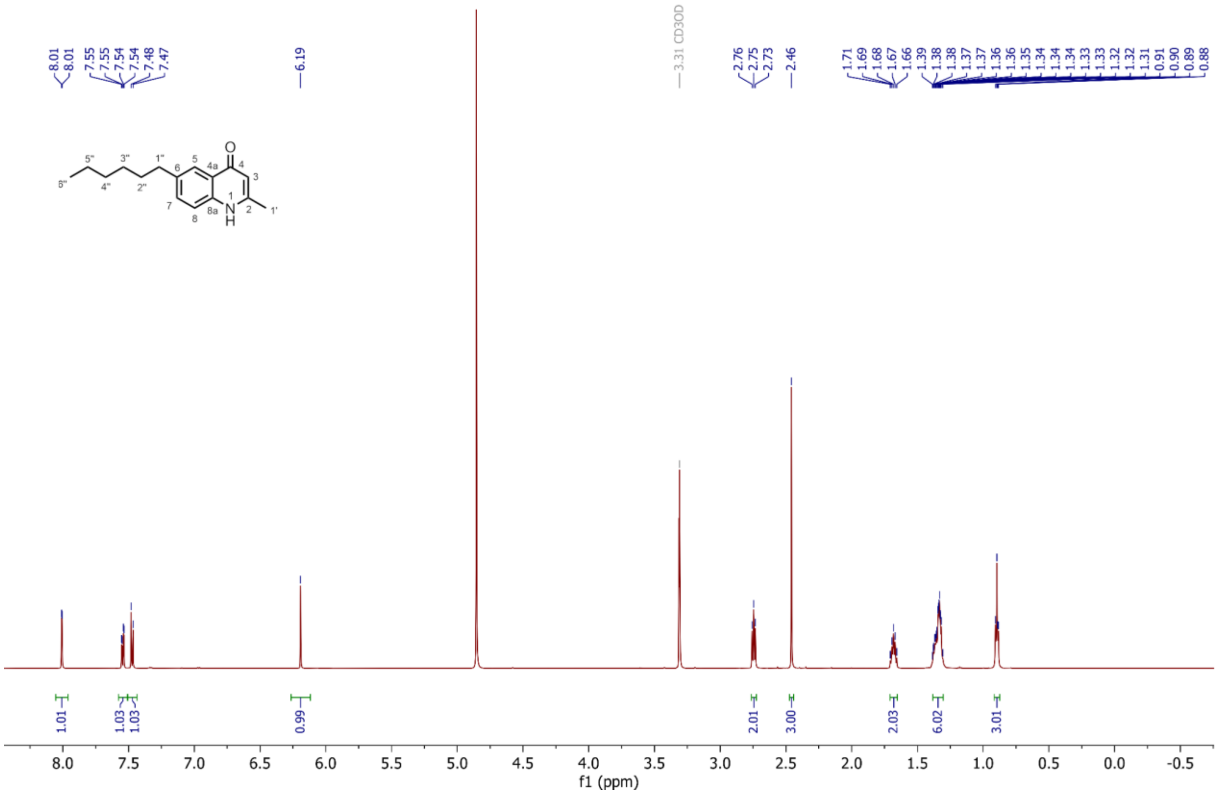

Figure S4. ^{1}H NMR spectrum (600 MHz, methanol-d_4) of 6-hexyl-2-methyl-1*H*-quinolin-4-one (**Yb**).

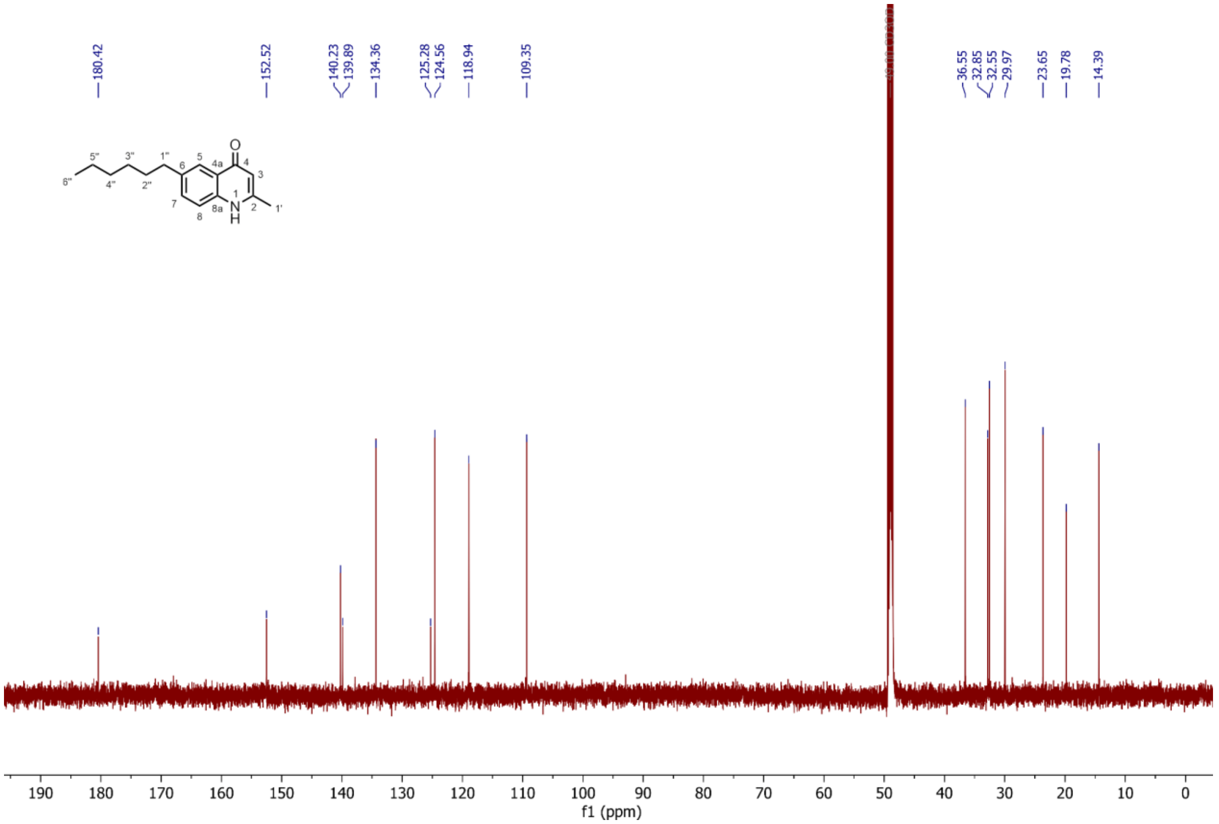

Figure S5. ^{1}H-decoupled ^{13}C NMR spectrum (150 MHz, methanol-d_4) of 6-hexyl-2-methyl-1*H*-quinolin-4-one (**Yb**).

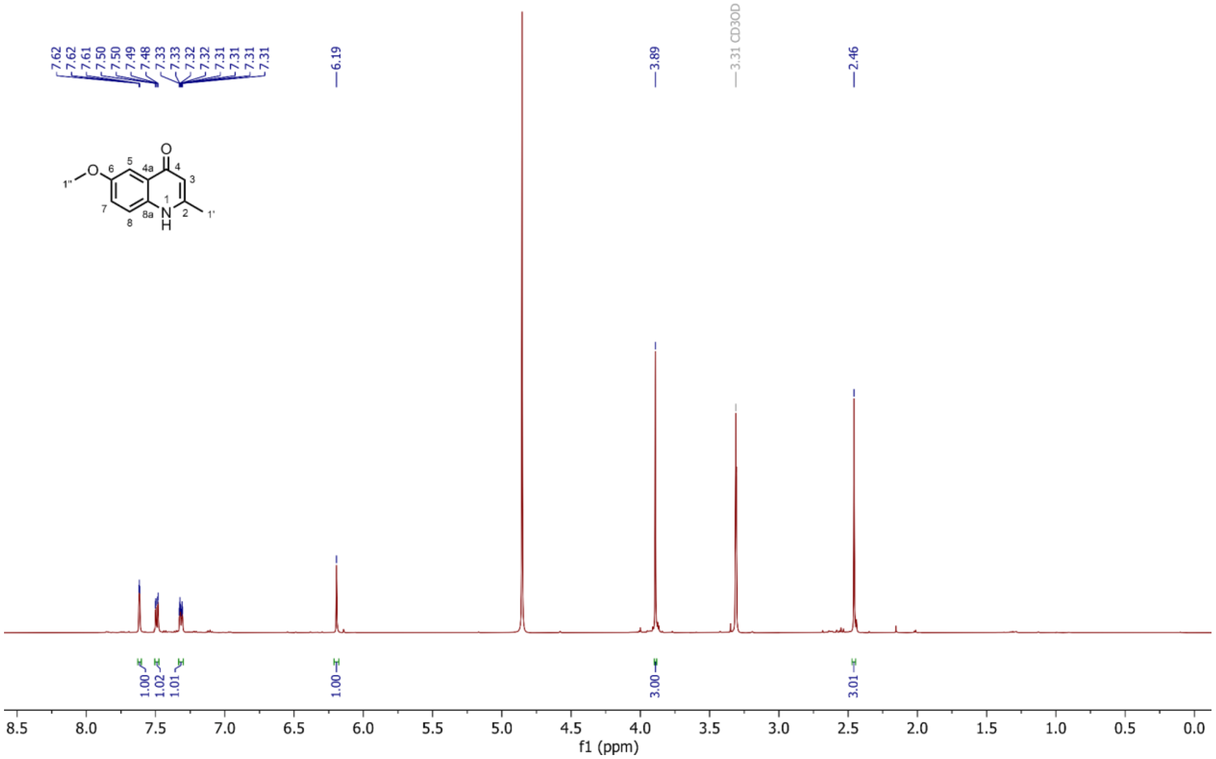

Figure S6. ^{1}H NMR spectrum (600 MHz, methanol-d_4) of 6-methoxy-2-methyl-1*H*-quinolin-4-one (**Yc**).

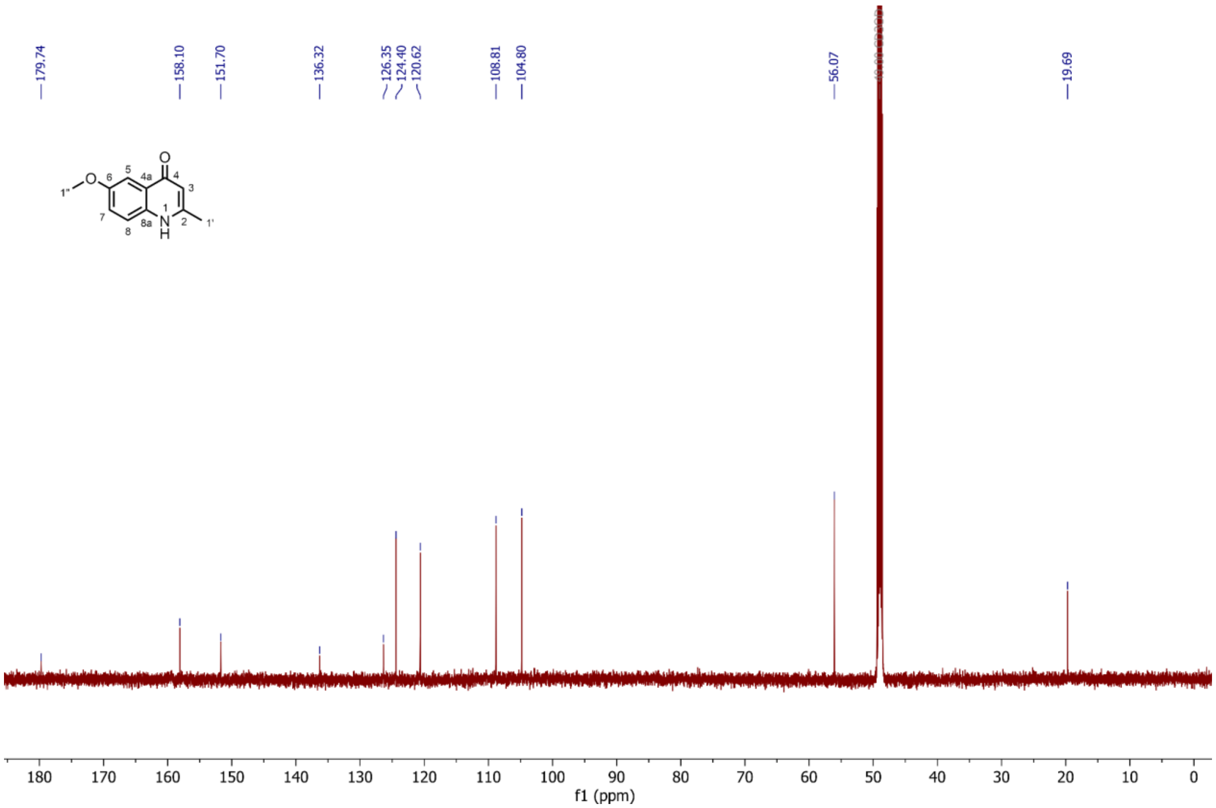

Figure S7. ^{1}H-decoupled ^{13}C NMR spectrum (150 MHz, methanol-d_4) of 6-methoxy-2-methyl-1*H*-quinolin-4-one (**Yc**).

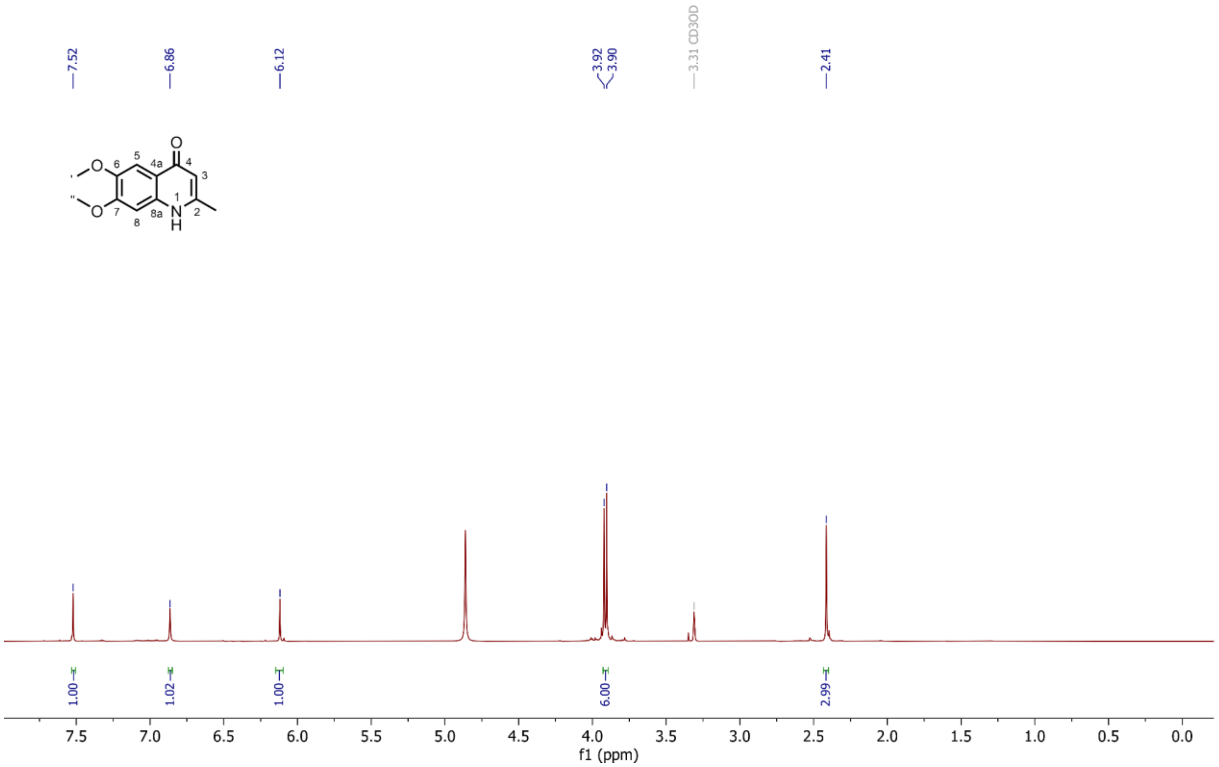

Figure S8. ^{1}H NMR spectrum (600 MHz, methanol-d_4) of 6,7-dimethoxy-2-methyl-1*H*-quinolin-4-one (**Yd**).

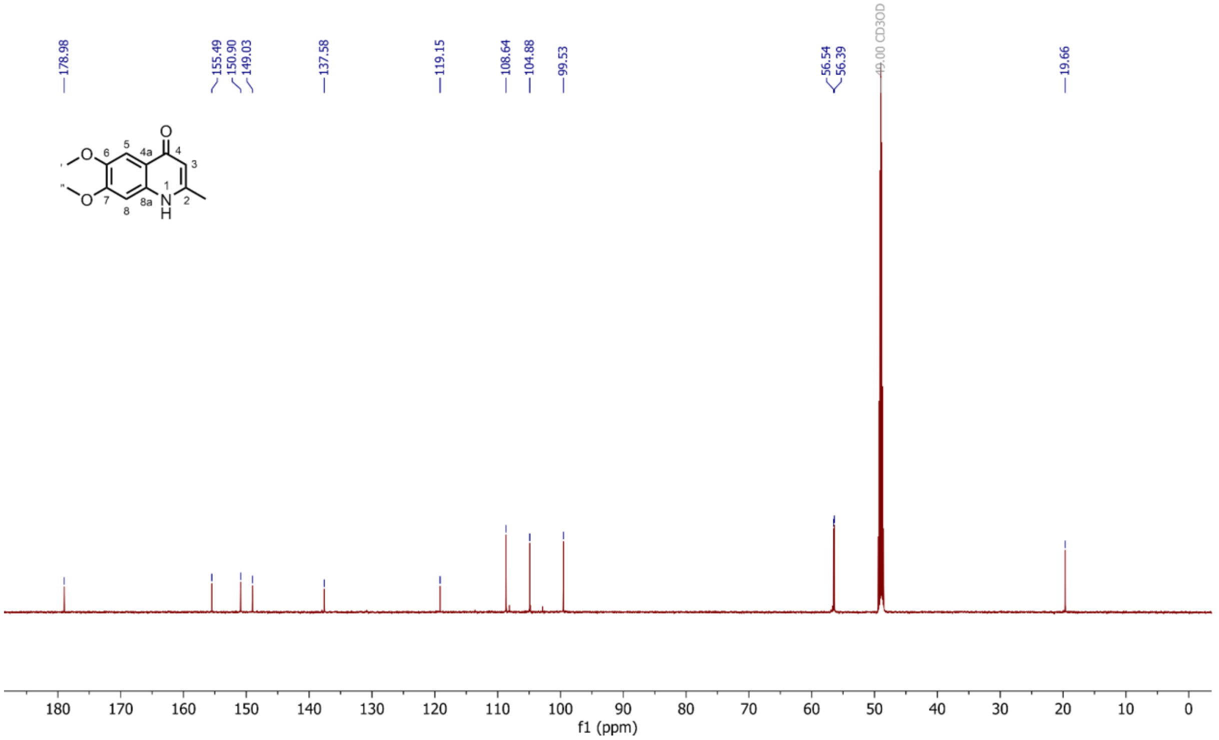

Figure S9. ^{1}H-decoupled ^{13}C NMR spectrum (150 MHz, methanol-d_4) of 6,7-dimethoxy-2-methyl-1*H*-quinolin-4-one (**Yd**).

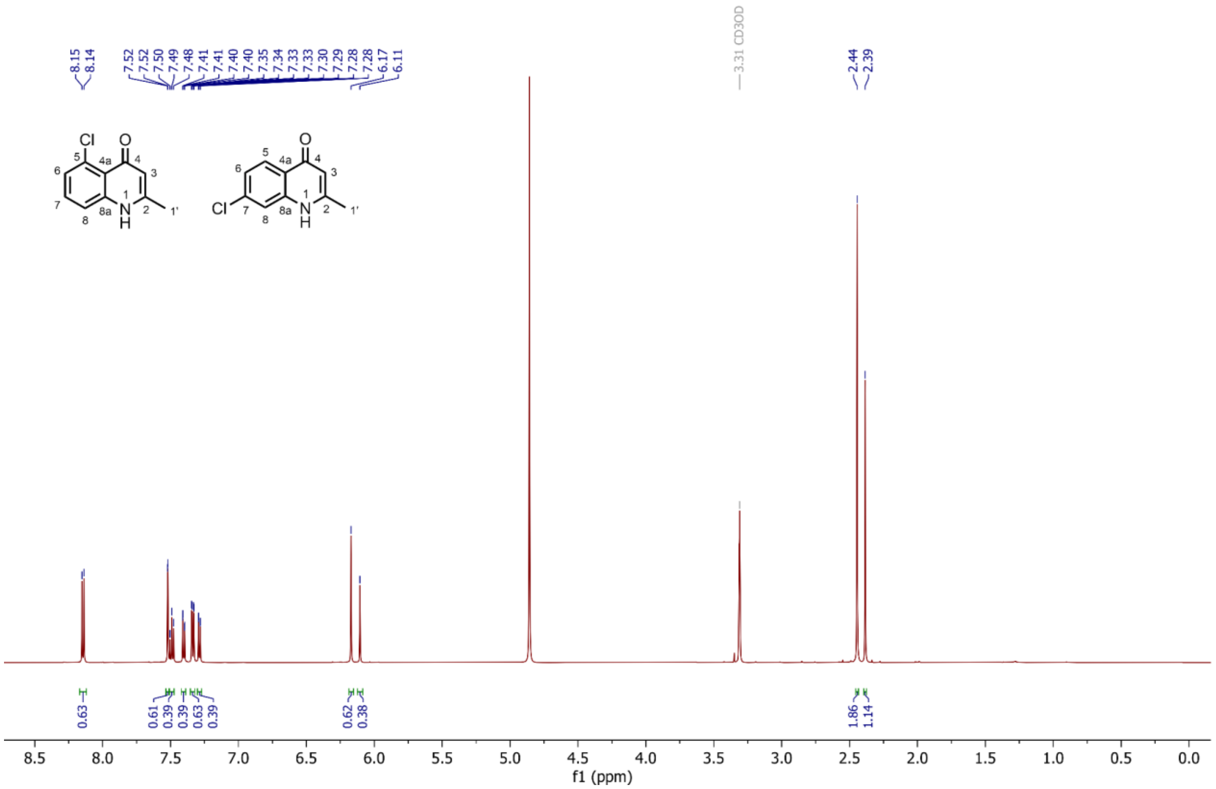

Figure S10. ^{1}H NMR spectrum (600 MHz, methanol-d_4) of a mixture of 7-chloro-2-methyl-1*H*-quinolin-4-one (**Ye**) and 5-chloro-2-methyl-1*H*-quinolin-4-one (**Ye′**).

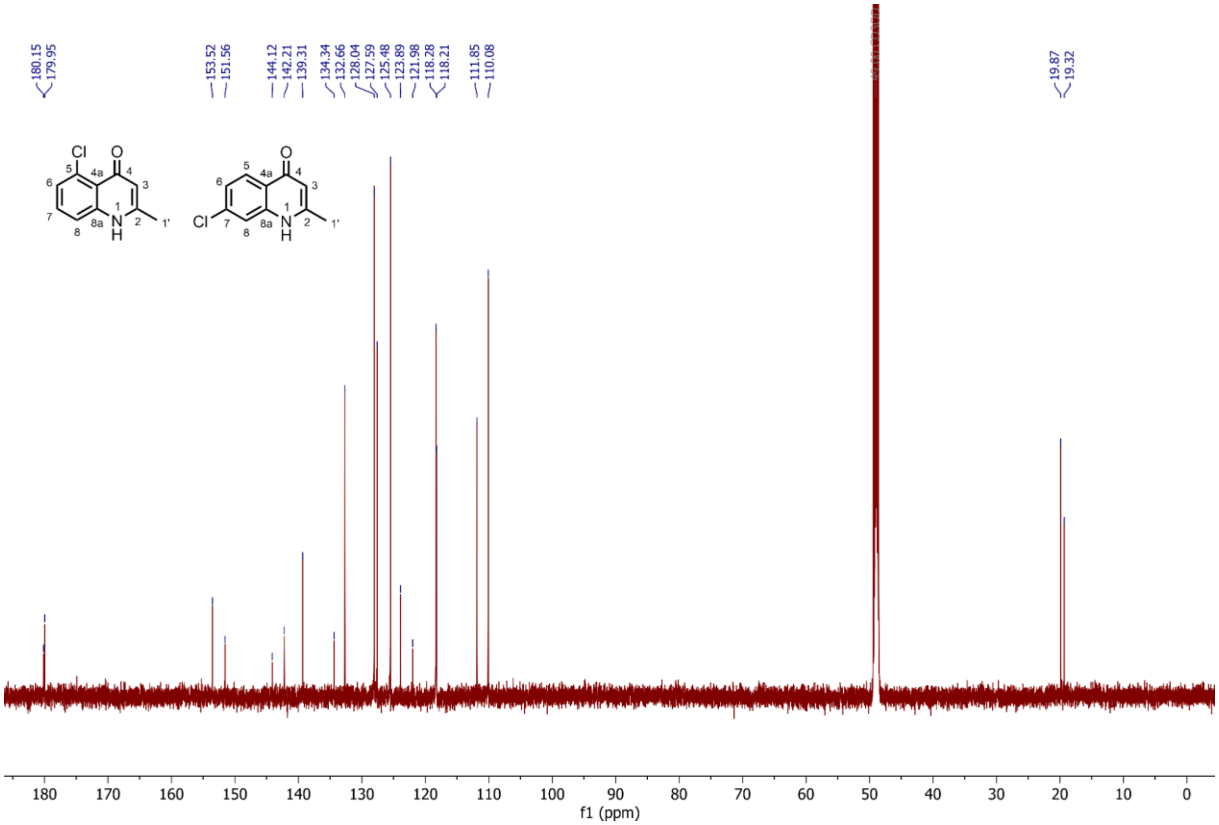

Figure S11. ^{1}H-decoupled ^{13}C NMR spectrum (150 MHz, methanol-d_4) of a mixture of **7**-chloro-2-methyl-1*H*-quinolin-4-one (**Ye**) and 5-chloro-2-methyl-1*H*-quinolin-4-one (**Ye′**).

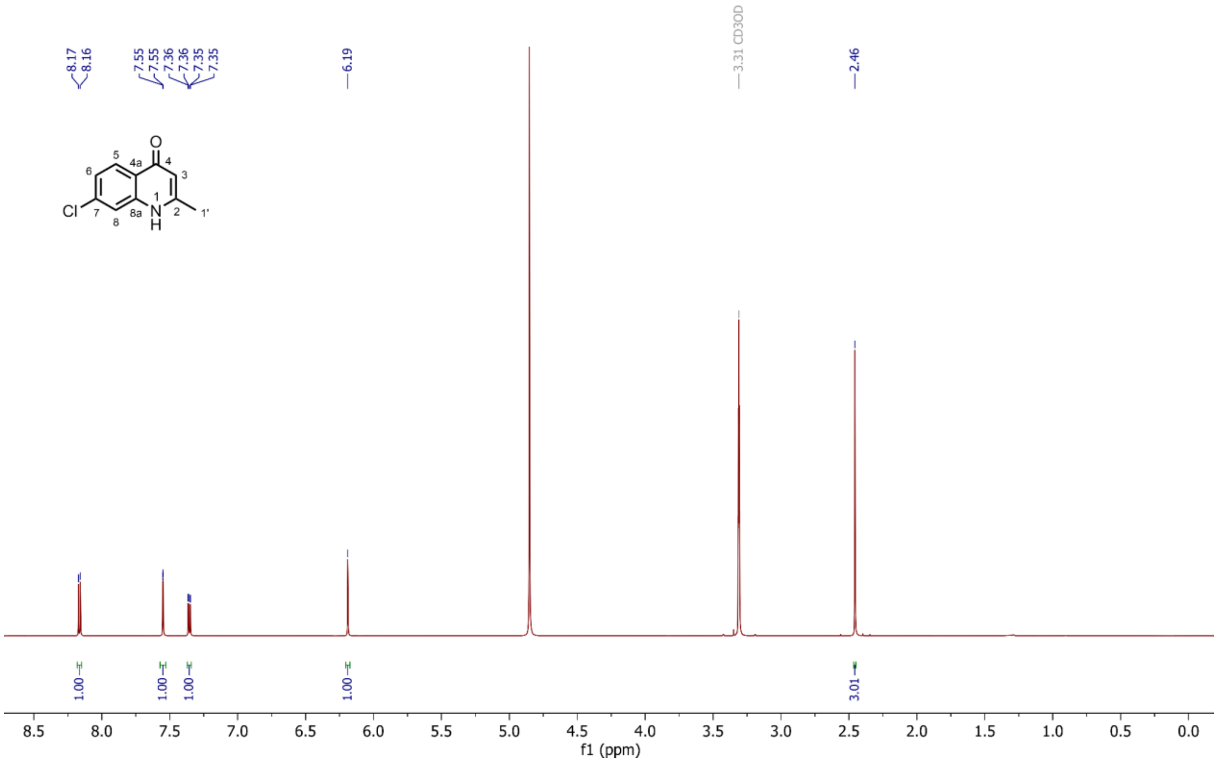

Figure S12. ^{1}H NMR spectrum (600 MHz, methanol-d_4) of 7-chloro-2-methyl-1*H*-quinolin-4-one (**Ye**).

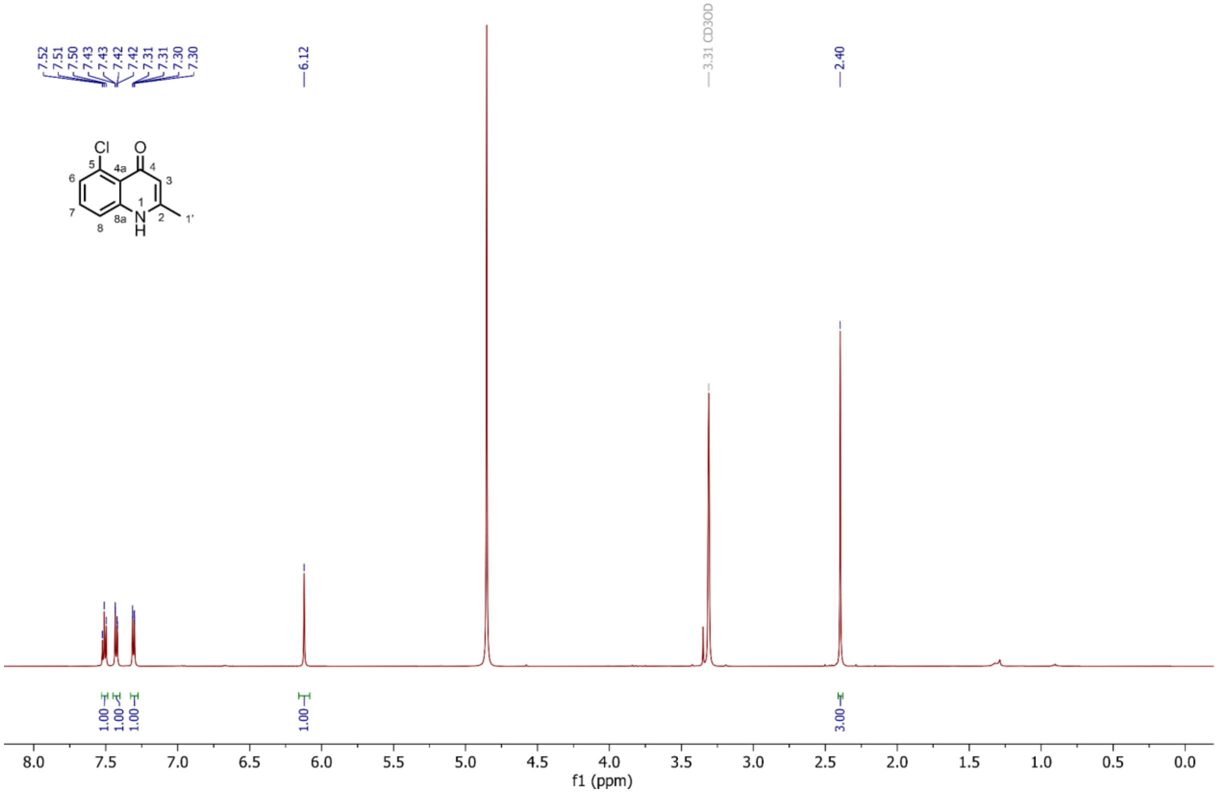

Figure S13. ^{1}H NMR spectrum (600 MHz, methanol-d_4) of **Ye'**.

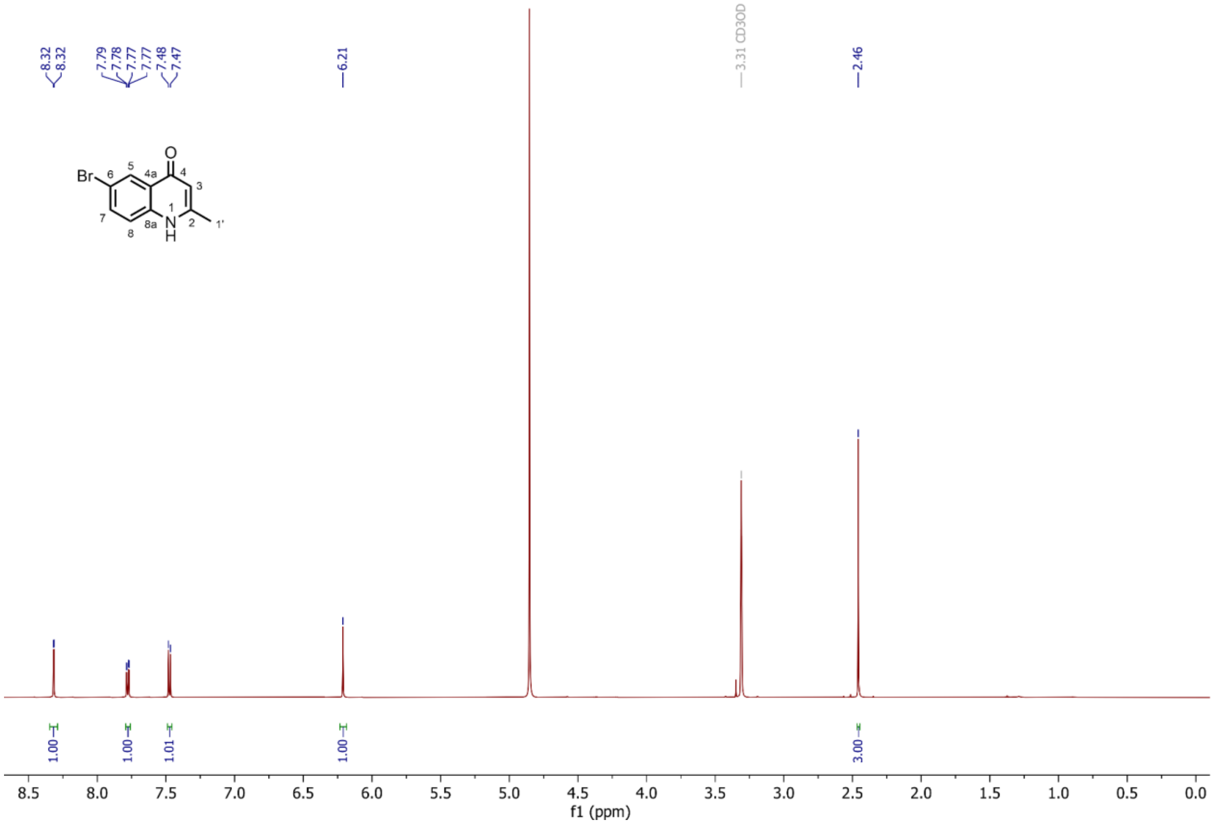

Figure S14. ^{1}H NMR spectrum (600 MHz, methanol-d_4) of 6-bromo-2-methyl-1*H*-quinolin-4-one (**Yg**).

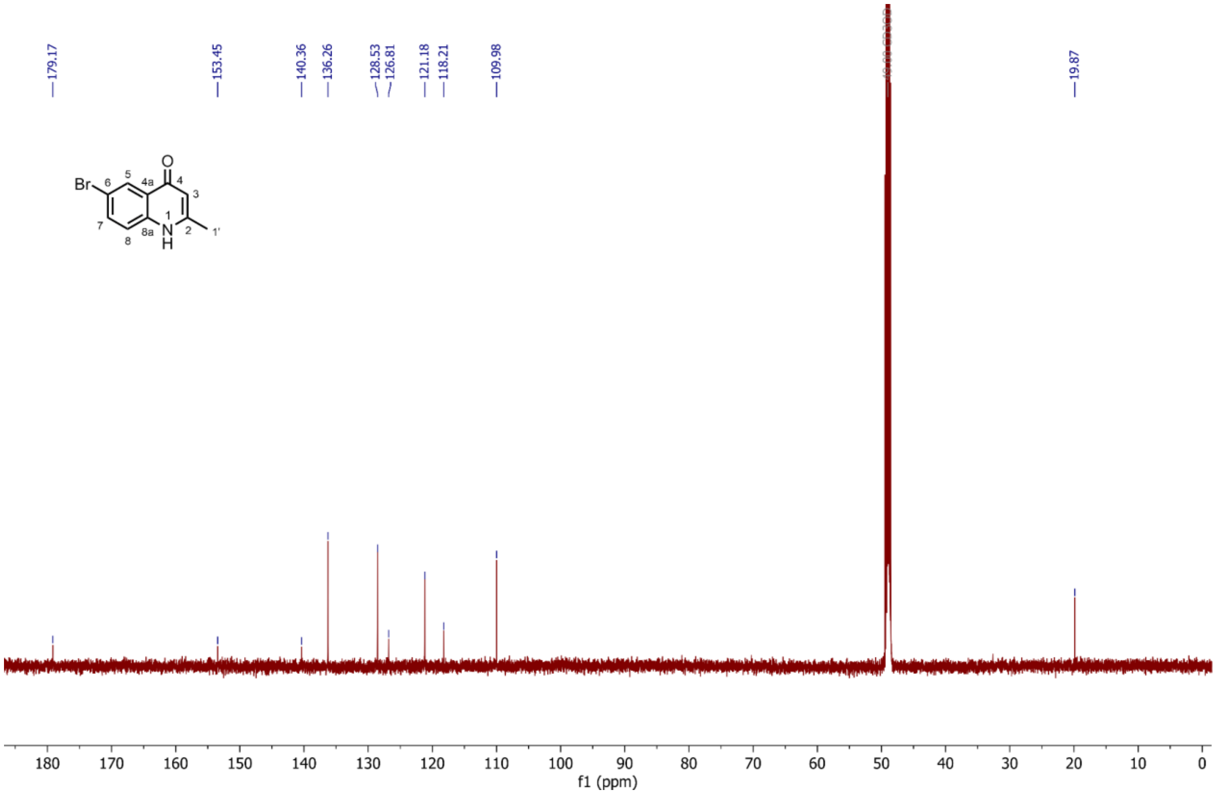

Figure S15. ^{1}H-decoupled ^{13}C NMR spectrum (150 MHz, methanol-d_4) of 6-bromo-2-methyl-1*H*-quinolin-4-one (**Yg**).

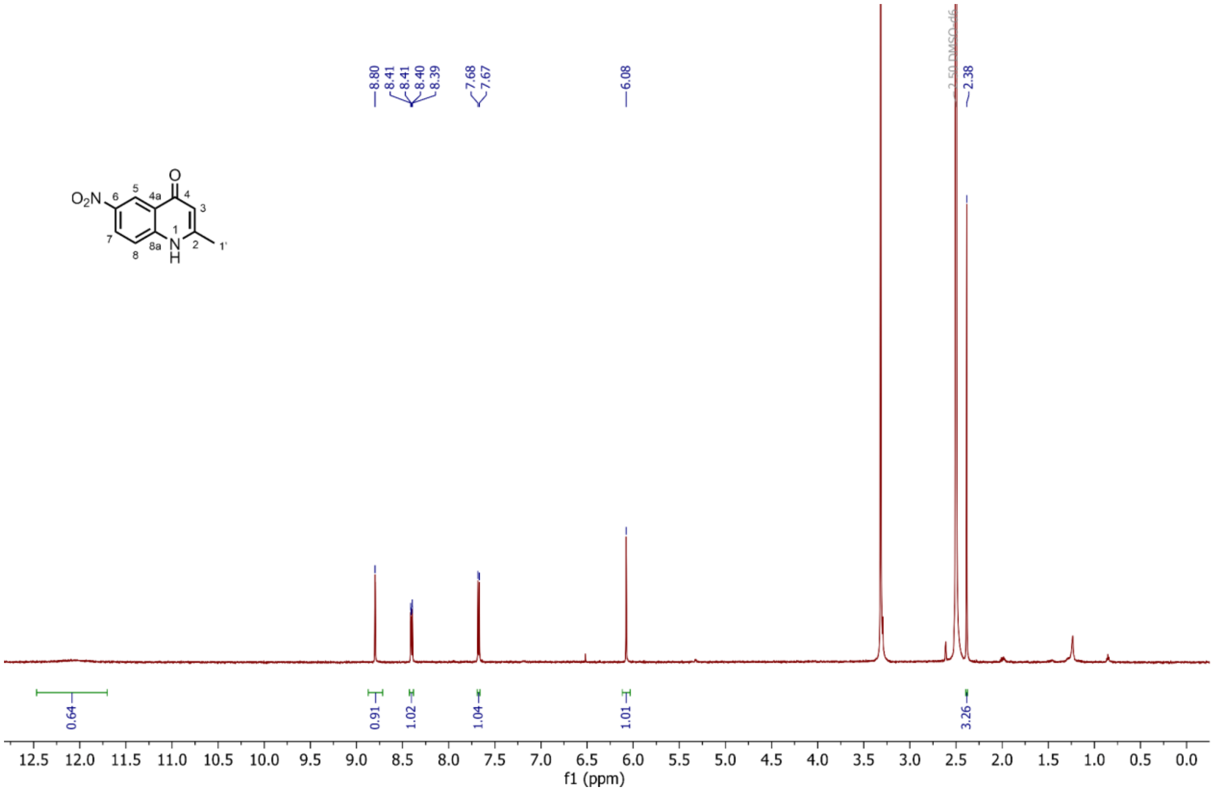

Figure S16. ^{1}H NMR spectrum (600 MHz, DMSO-d_6) of 2-methyl-6-nitro-1*H*-quinolin-4-one (**Yh**).

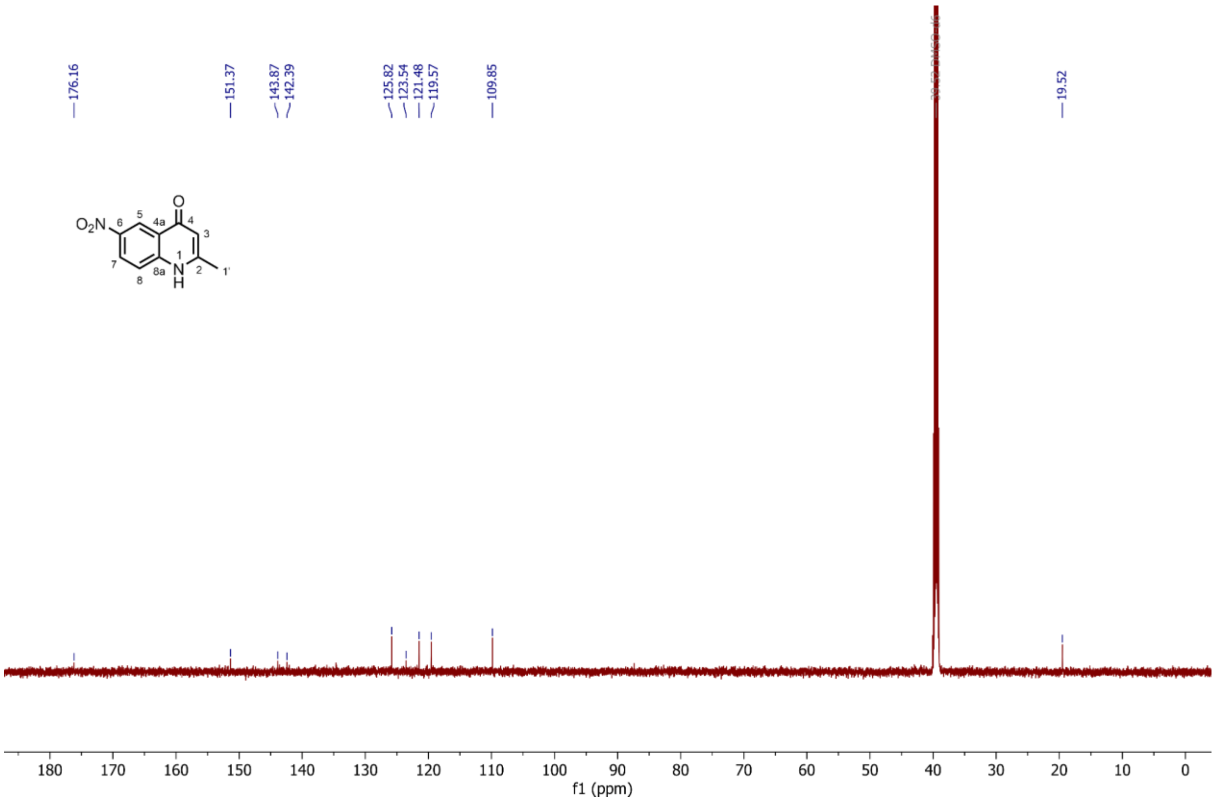

Figure S17. ^{1}H-decoupled ^{13}C NMR spectrum (150 MHz, DMSO-d_6) of 2-methyl-6-nitro-1*H*-quinolin-4-one (**Yh**).

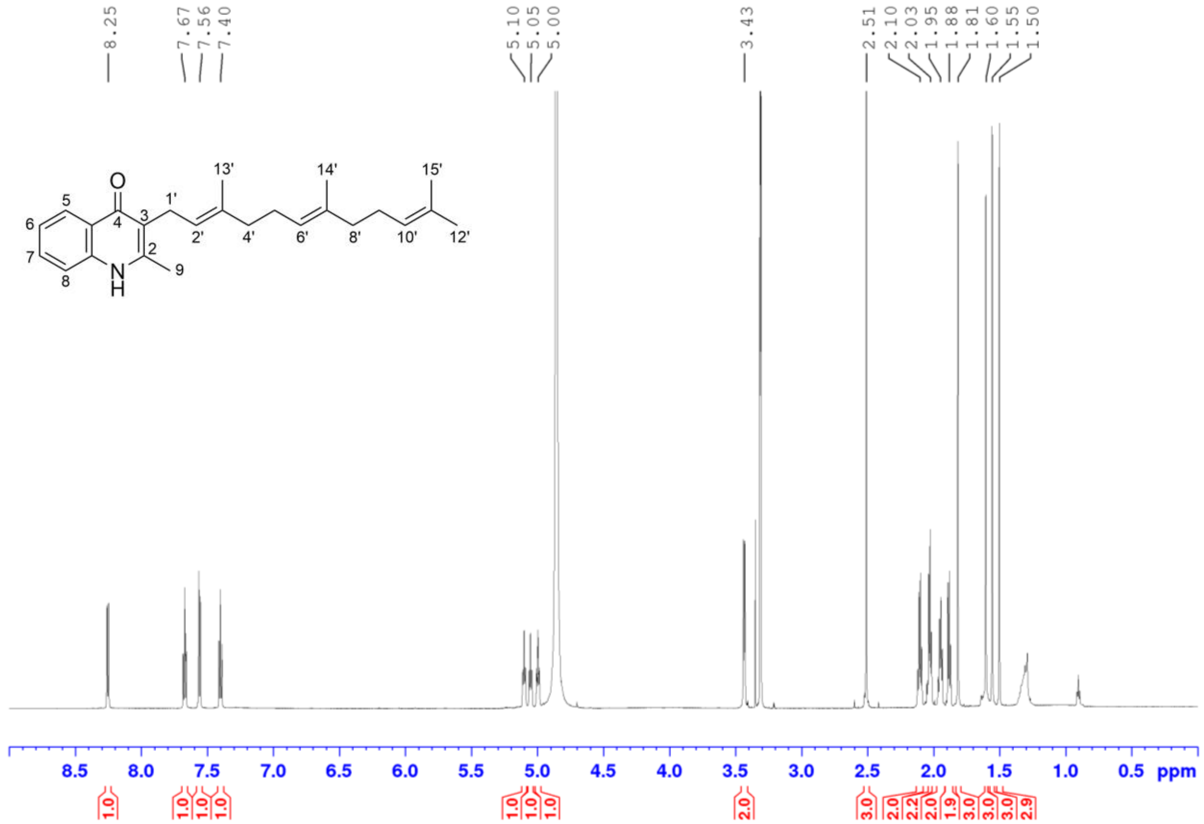

Figure S18. ^{1}H NMR spectrum (700 MHz, methanol-d_4) of **1**.

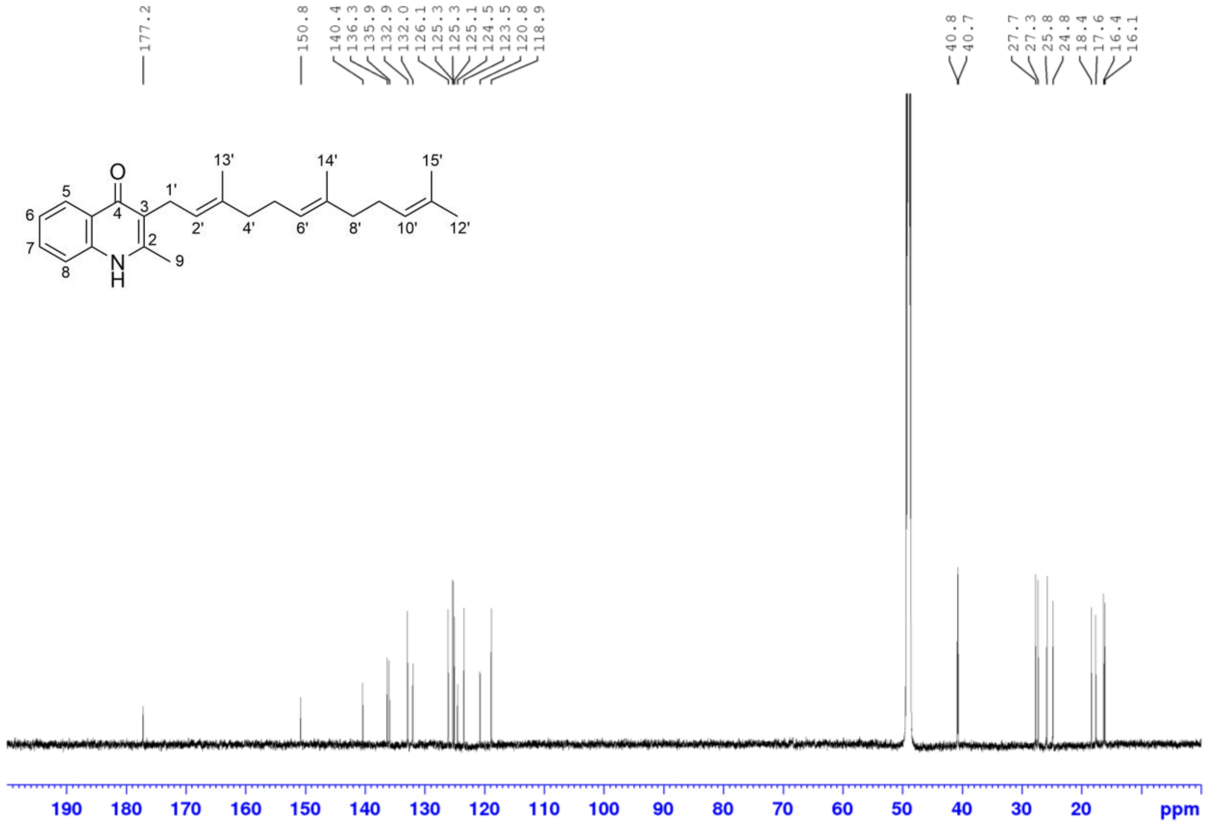

Figure S19. ^{1}H-decoupled ^{13}C NMR spectrum (175 MHz, methanol-d_4) of **1**.

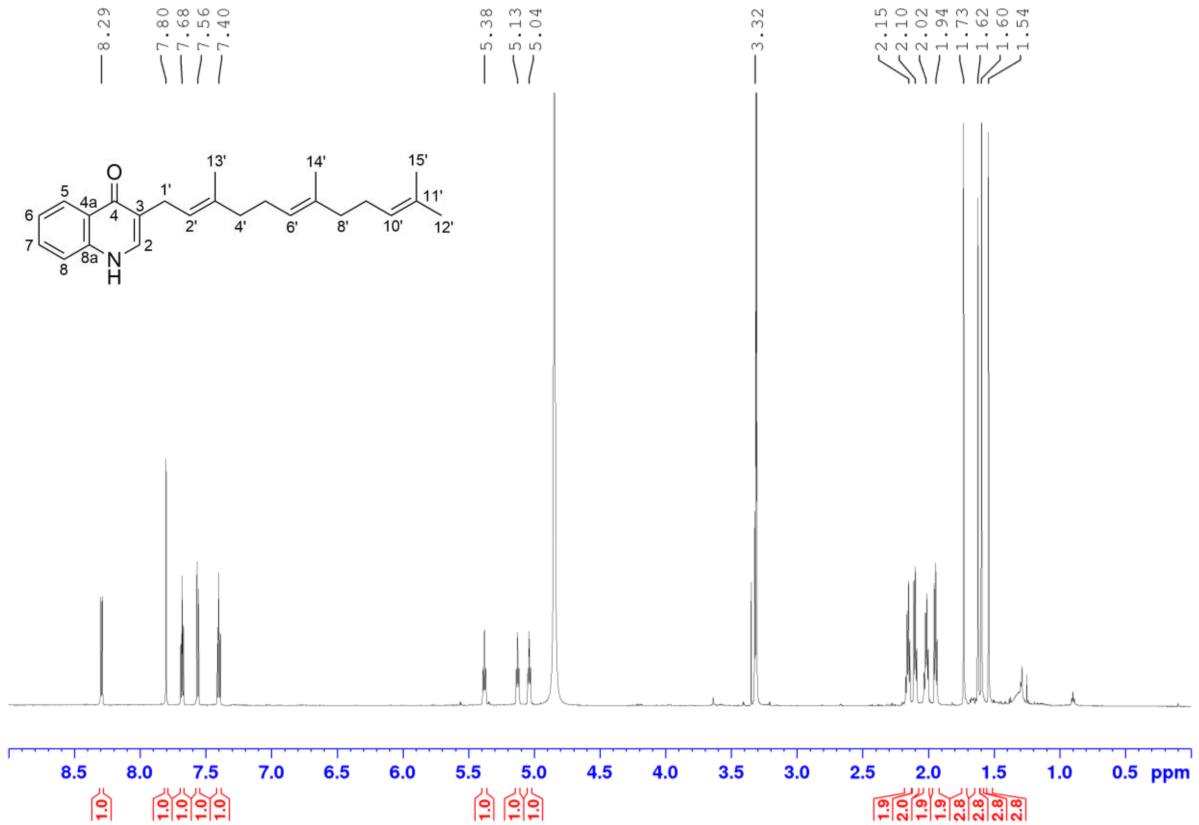

Figure S20. ^{1}H NMR spectrum (700 MHz, methanol-d_4) of **2**.

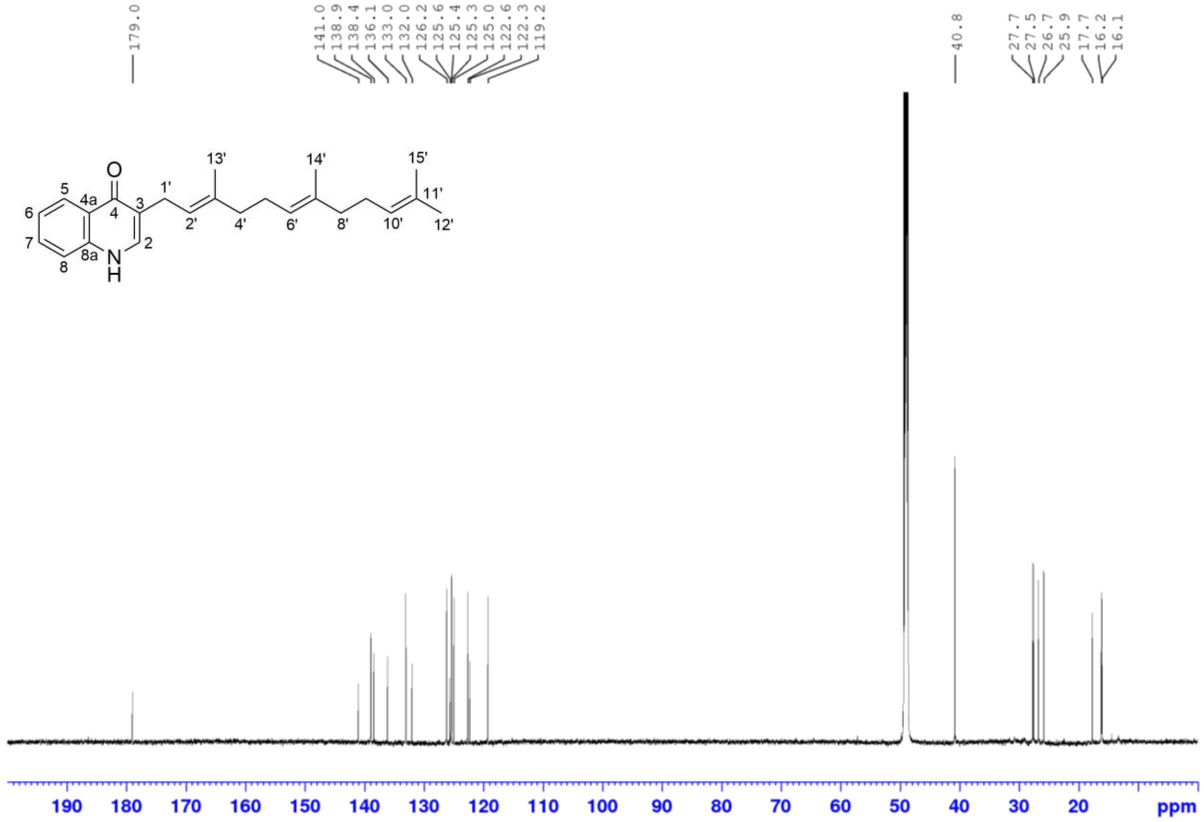

Figure S21. ^{1}H-decoupled ^{13}C NMR spectrum (175 MHz, methanol-d_4) of **2**.

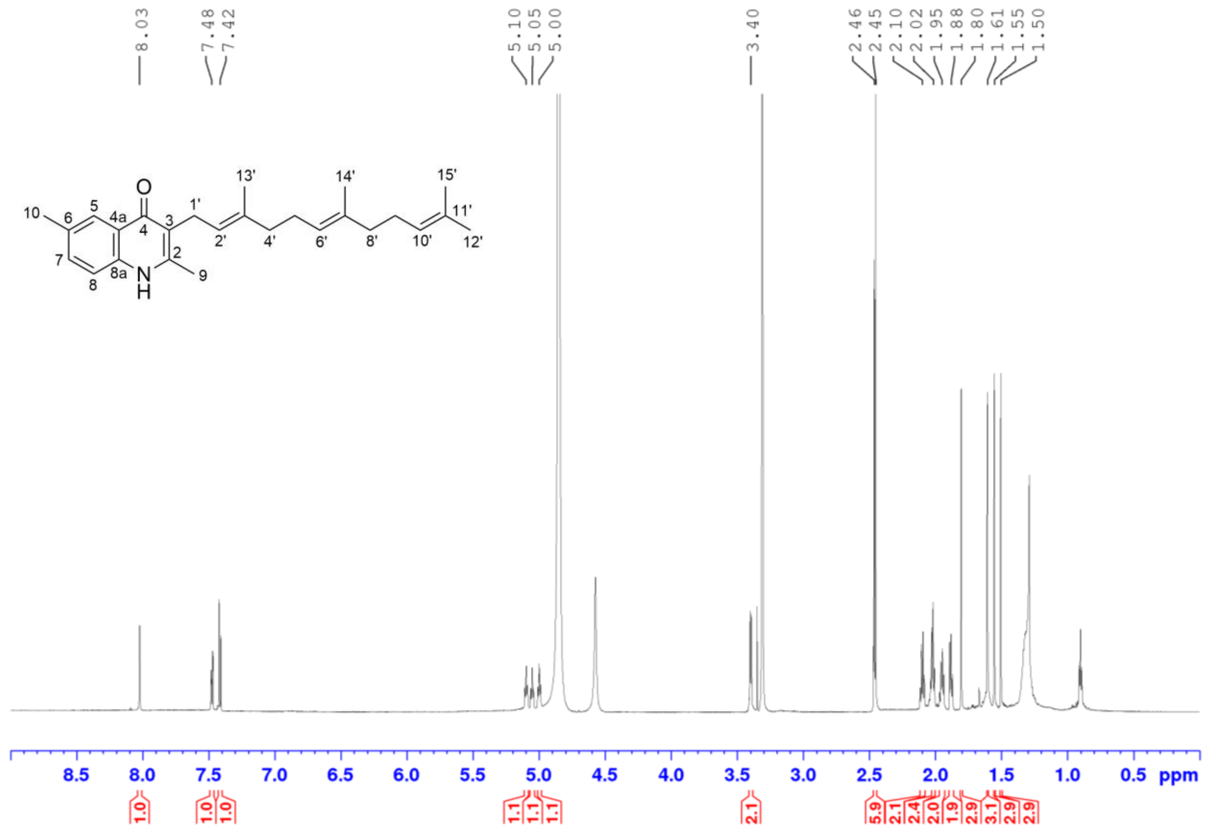

Figure S22. ^{1}H NMR spectrum (700 MHz, methanol-d_4) of **3**.

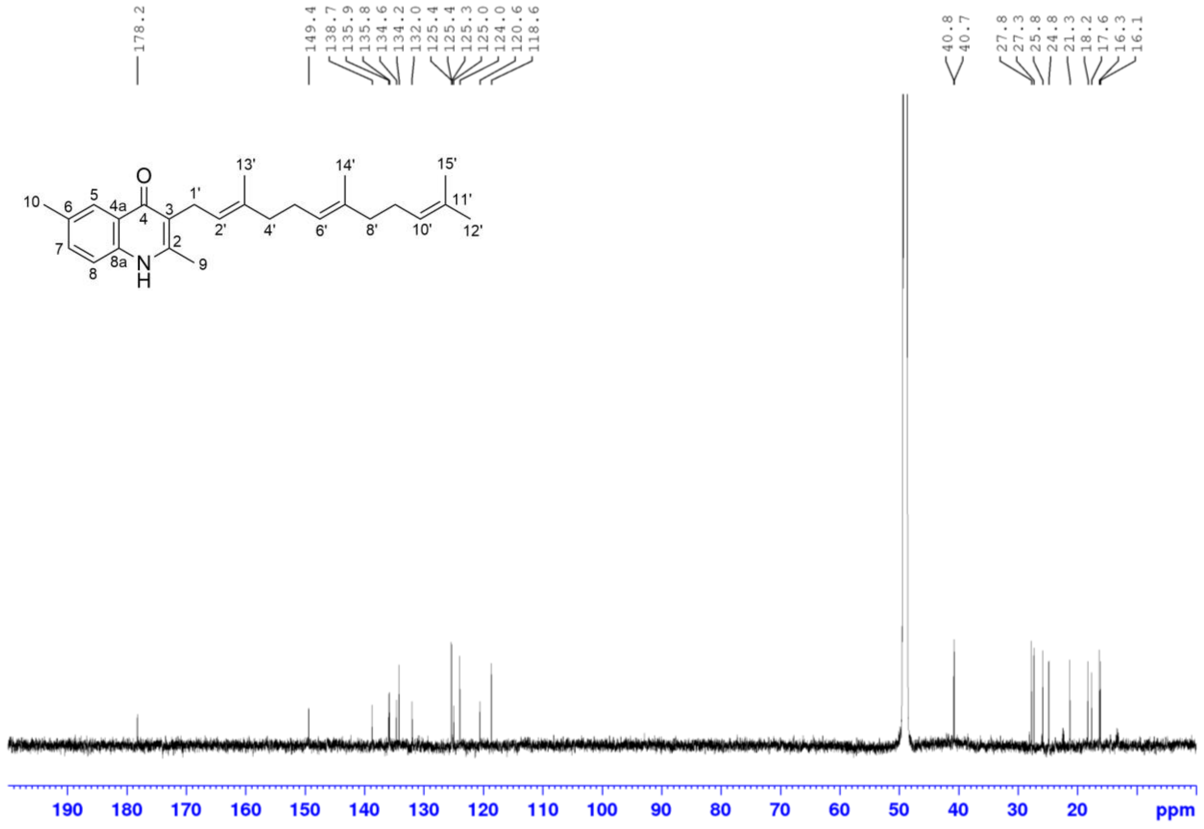

Figure S23. ^{1}H-decoupled ^{13}C NMR spectrum (175 MHz, methanol-d_4) of **3**.

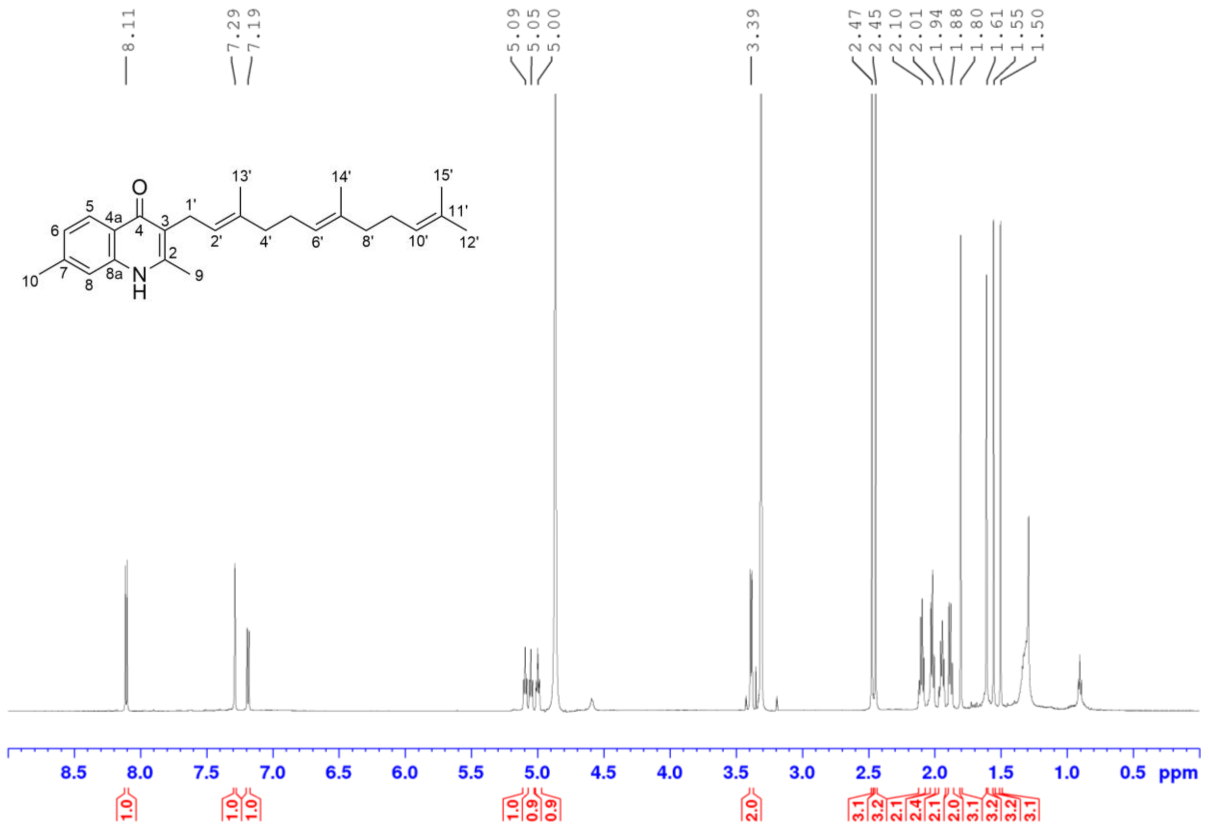

Figure S24. ^{1}H NMR spectrum (600 MHz, methanol-d_4) of **4**.

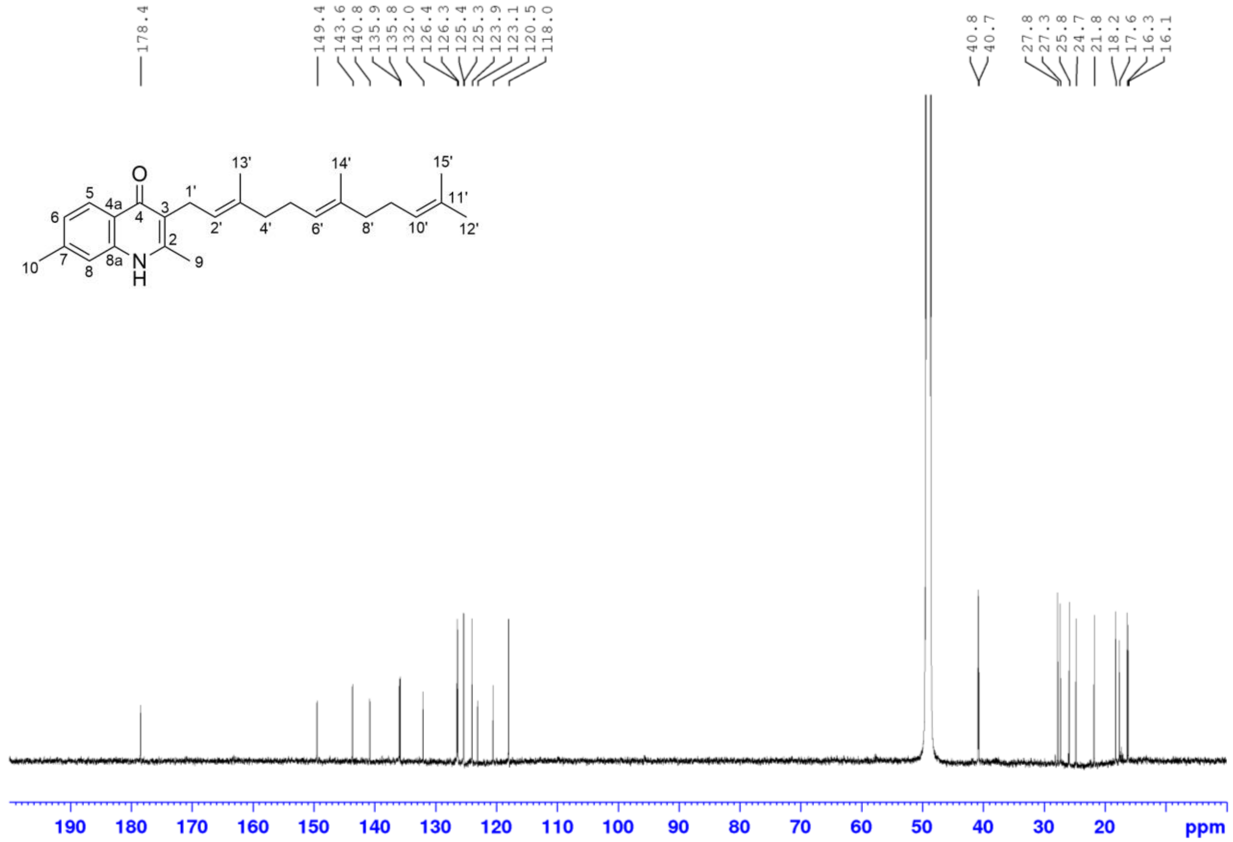

Figure S25. ^{1}H-decoupled ^{13}C NMR spectrum (150 MHz, methanol-d_4) of **4**.

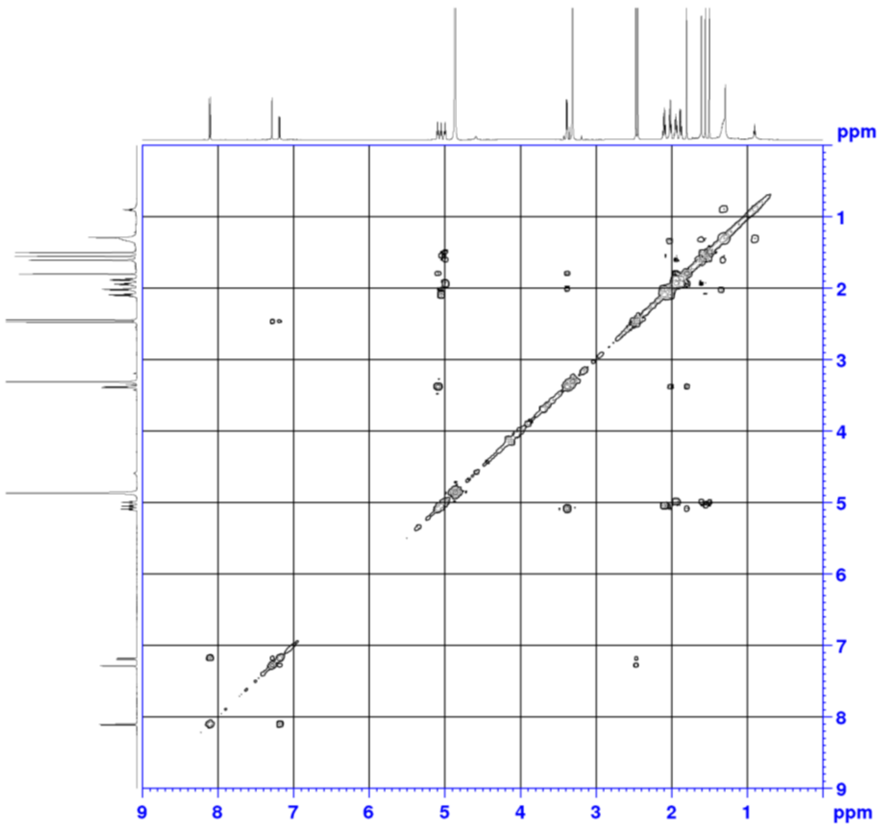

Figure S26. COSY spectrum (methanol-d_4) of **4**.

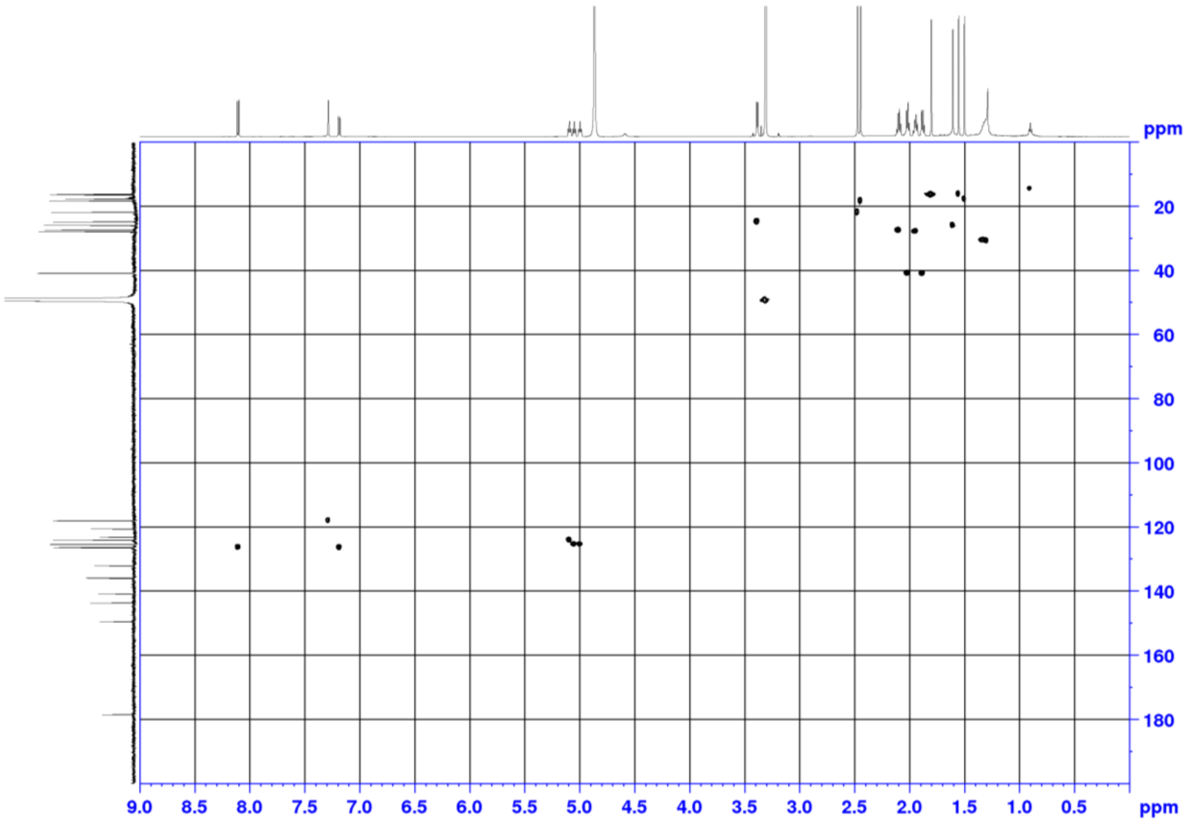

Figure S27. HSQC spectrum (methanol-d_4) of **4**.

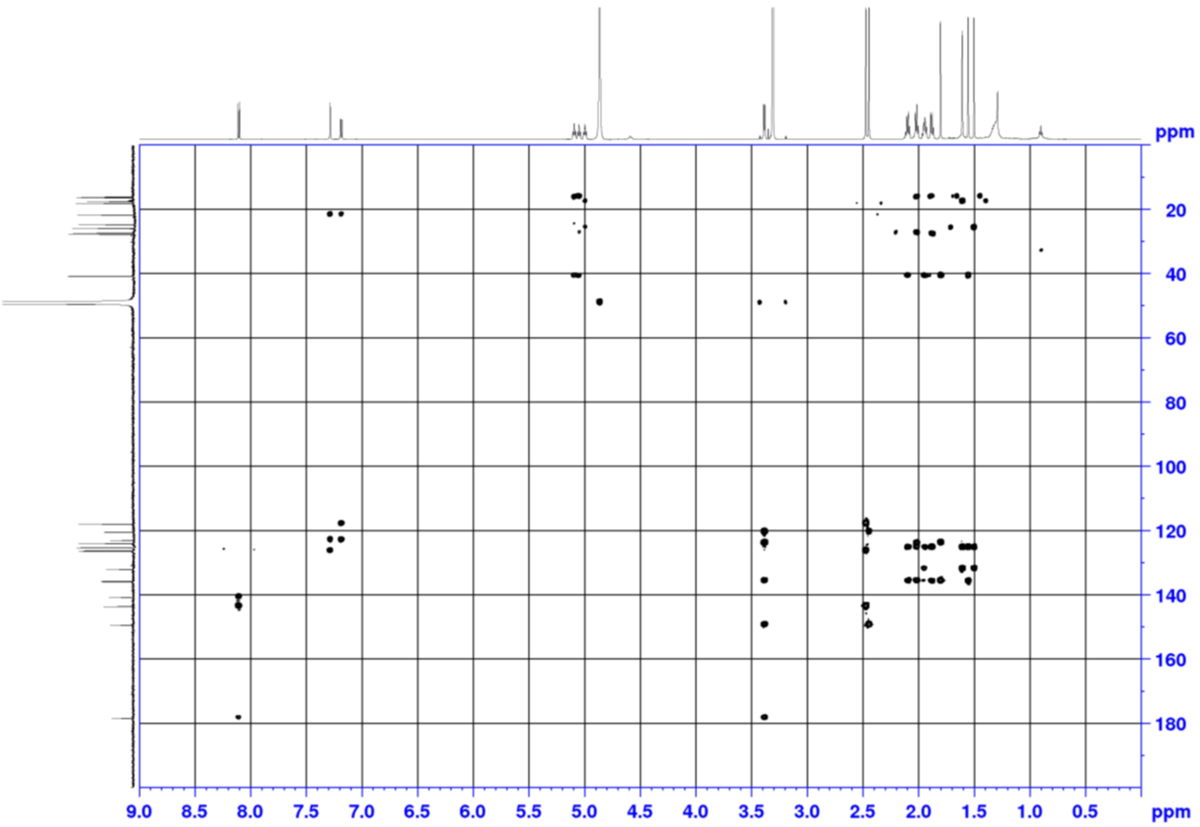

Figure S28. HMBC spectrum (methanol-d_4) of **4**.

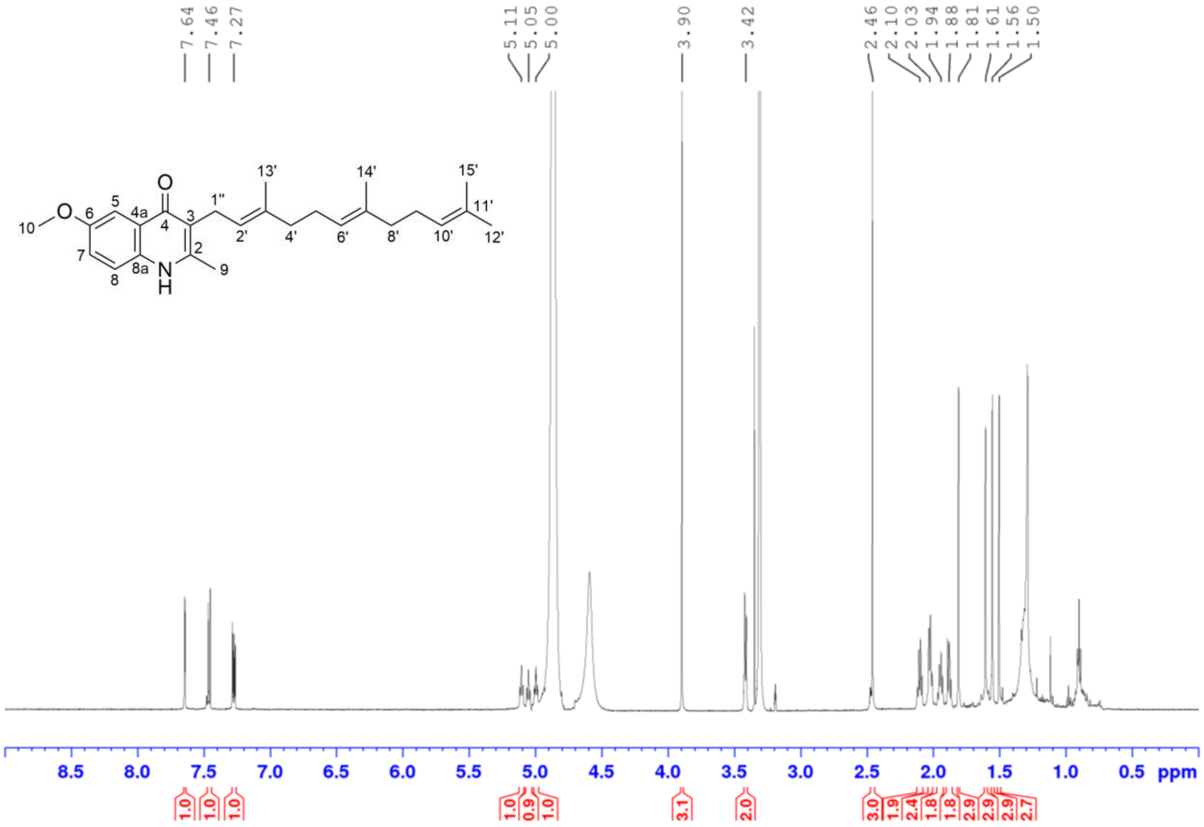

Figure S29. ^{1}H NMR spectrum (600 MHz, methanol-d_4) of **5**.

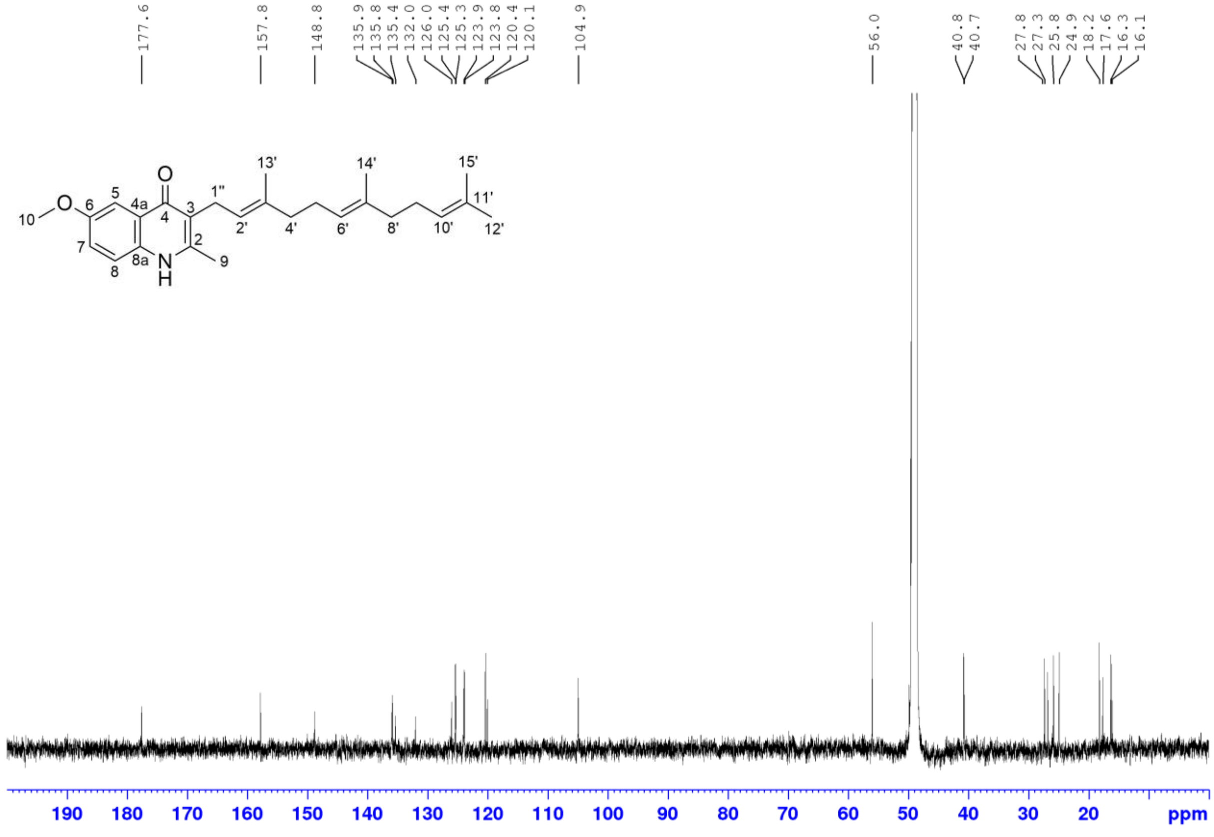

Figure S30. ^{1}H-decoupled ^{13}C NMR spectrum (150 MHz, methanol-d_4) of **5**.

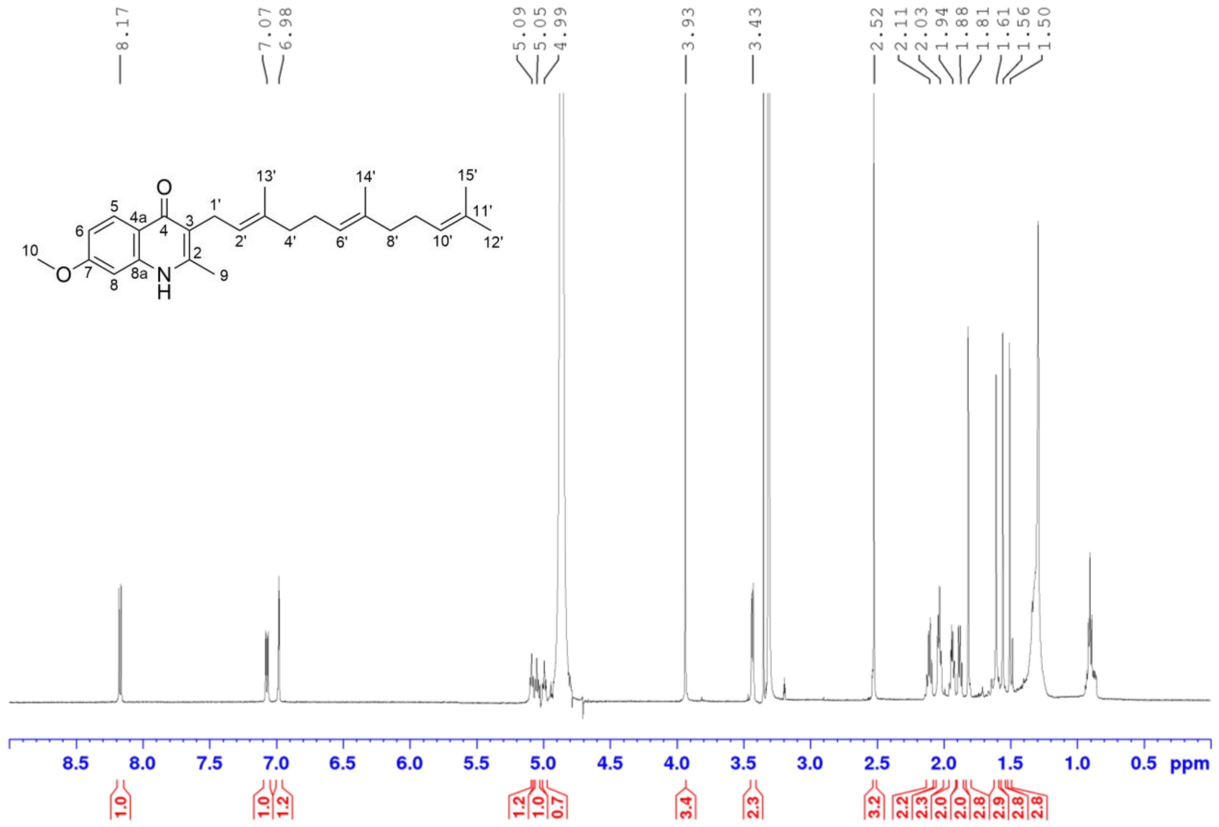

Figure S31. ^{1}H NMR spectrum (600 MHz, methanol-d_4) of **6**.

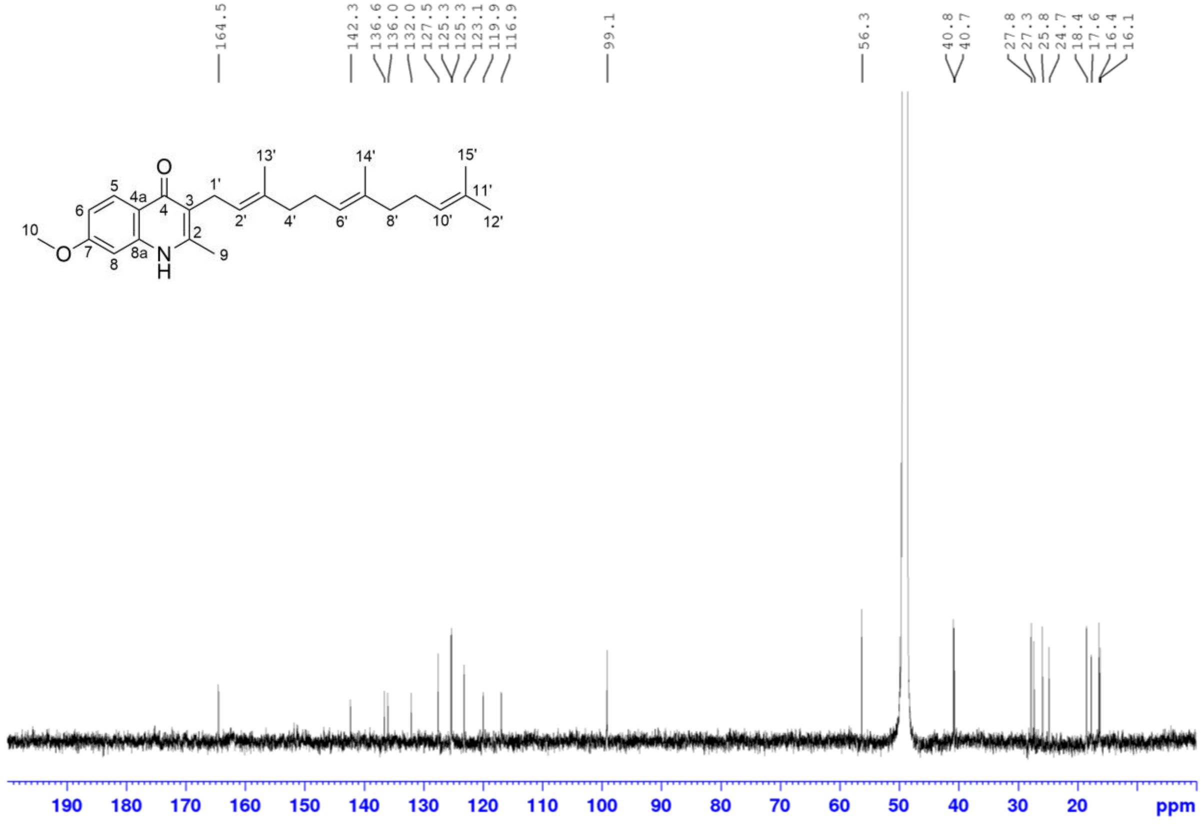

Figure S32. ^{1}H-decoupled ^{13}C NMR spectrum (150 MHz, methanol-d_4) of **6**.

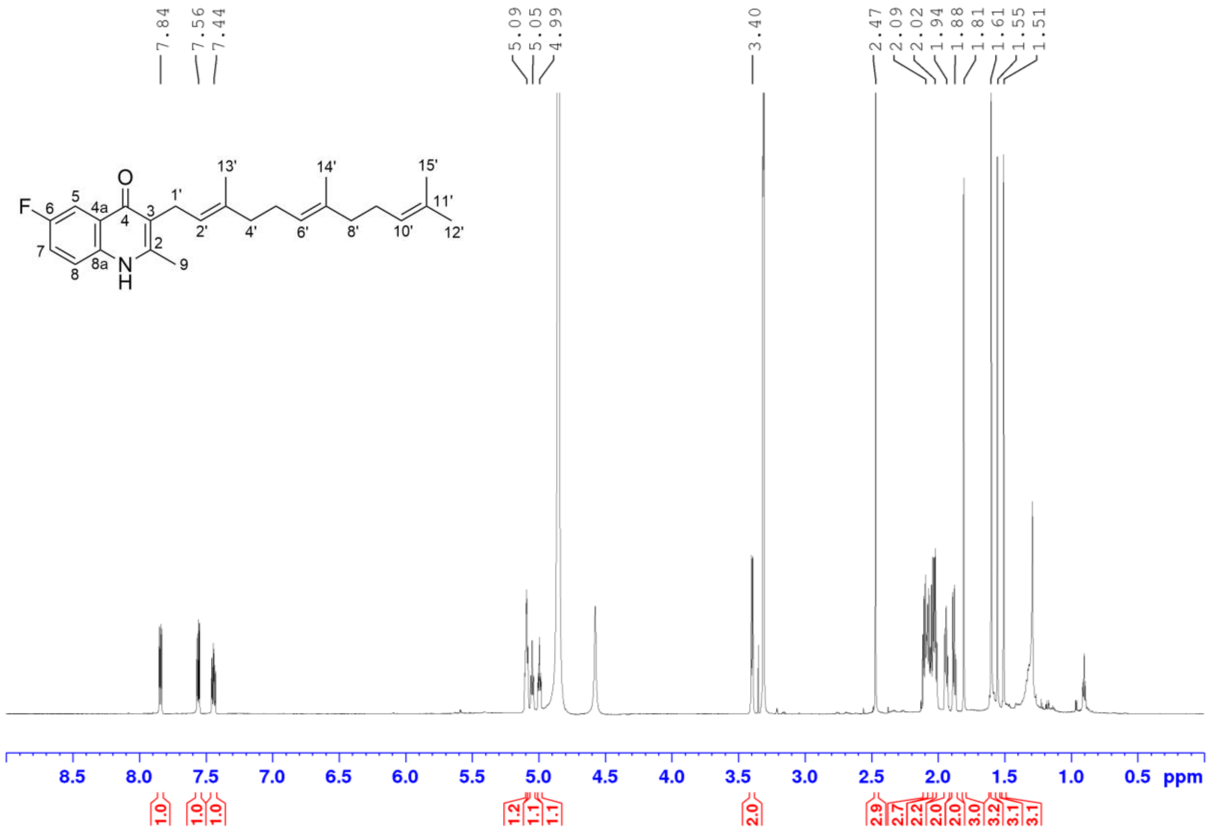

Figure S33. ^{1}H NMR spectrum (700 MHz, methanol-d_4) of **7**.

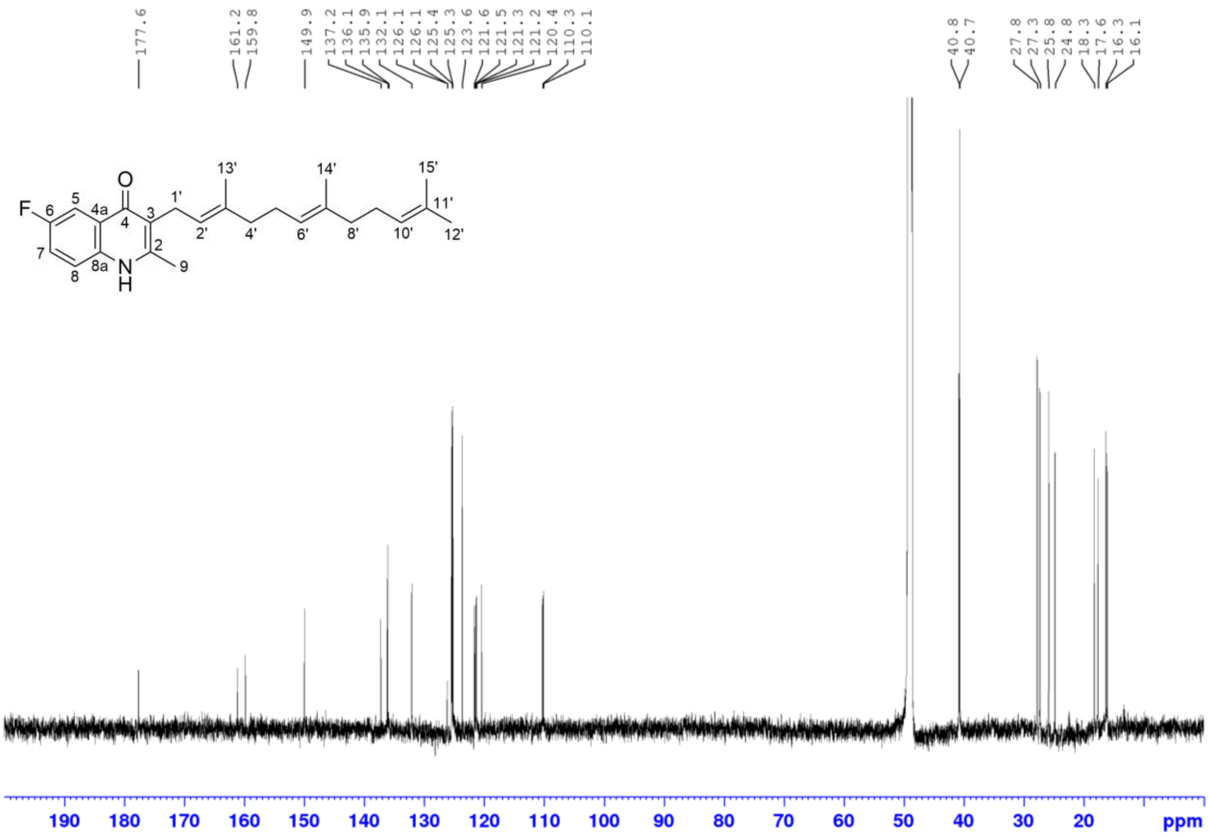

Figure S34. ^{1}H-decoupled ^{13}C NMR spectrum (175 MHz, methanol-d_4) of **7**.

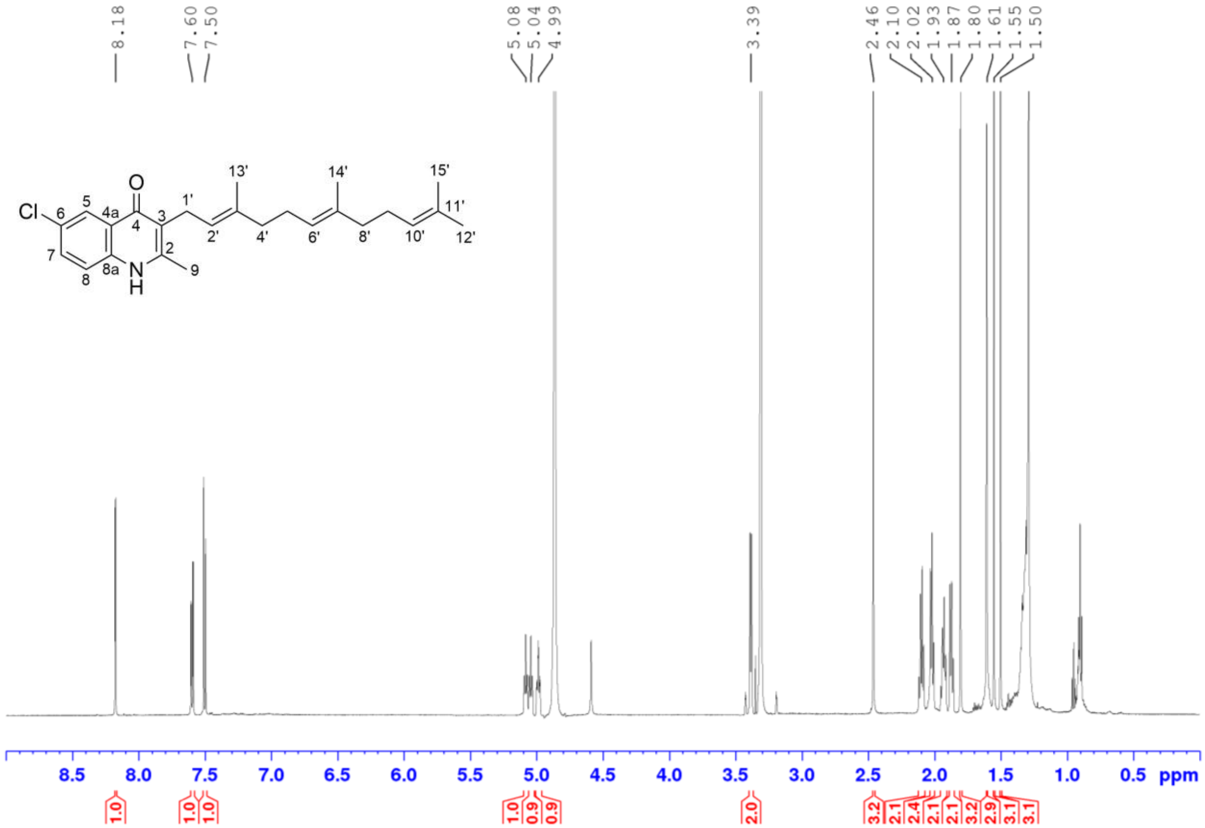

Figure S35. ^{1}H NMR spectrum (600 MHz, methanol-d_4) of **8**.

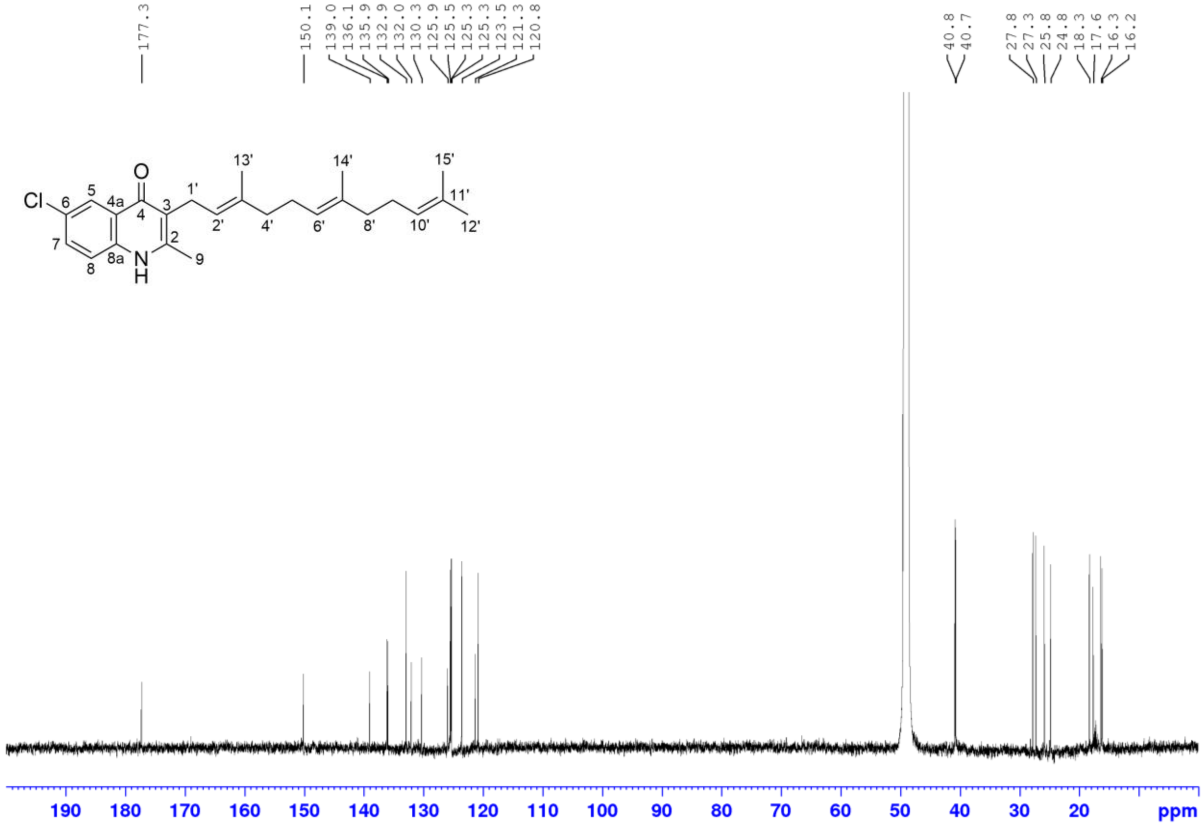

Figure S36. ^{1}H-decoupled ^{13}C NMR spectrum (150 MHz, methanol-d_4) of **8**.

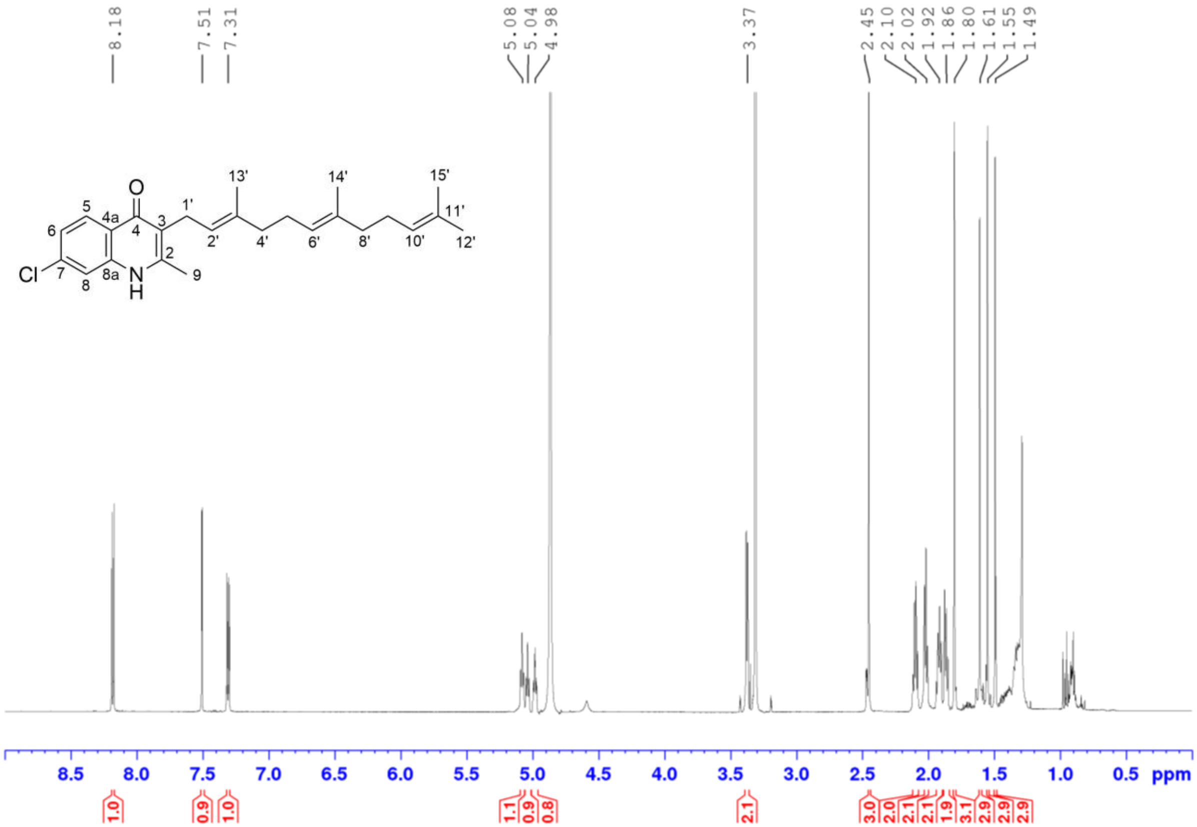

Figure S37. ^{1}H NMR spectrum (600 MHz, methanol-d_4) of **9**.

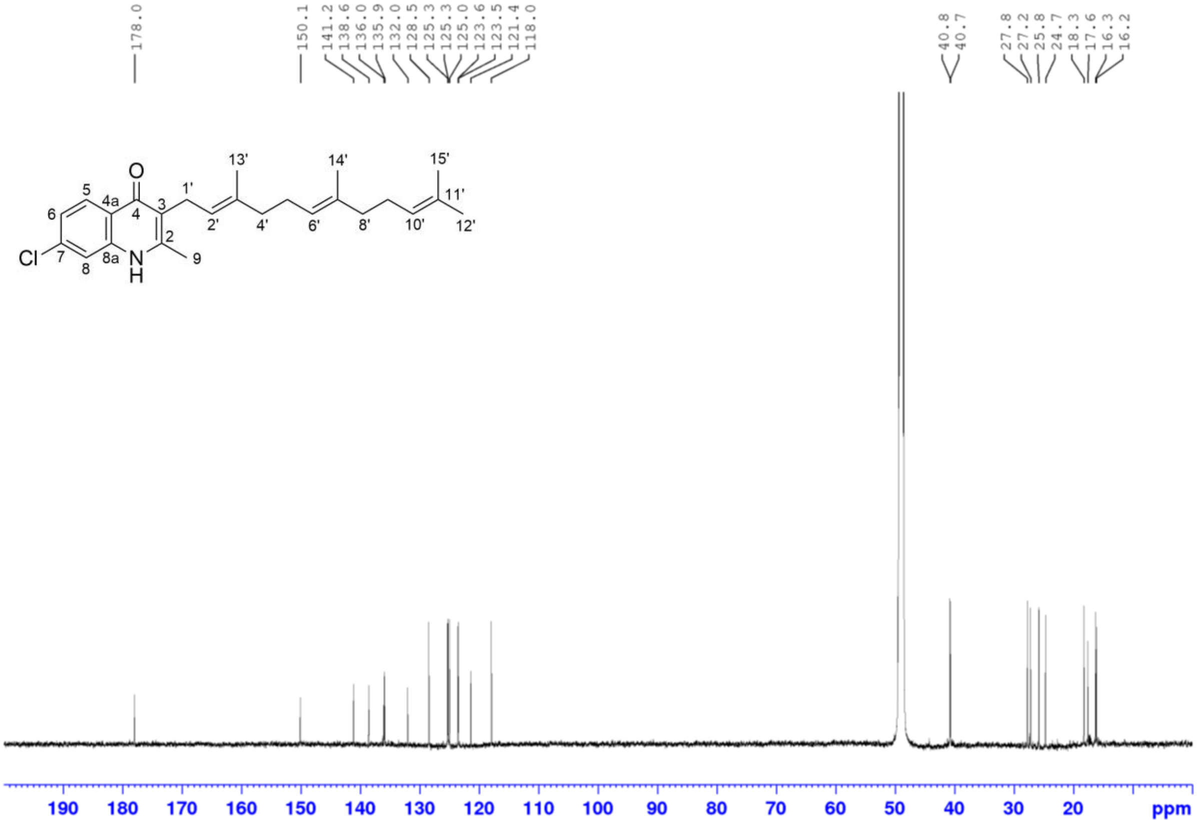

Figure S38. ^{1}H-decoupled ^{13}C NMR spectrum (150 MHz, methanol-d_4) of **9**.

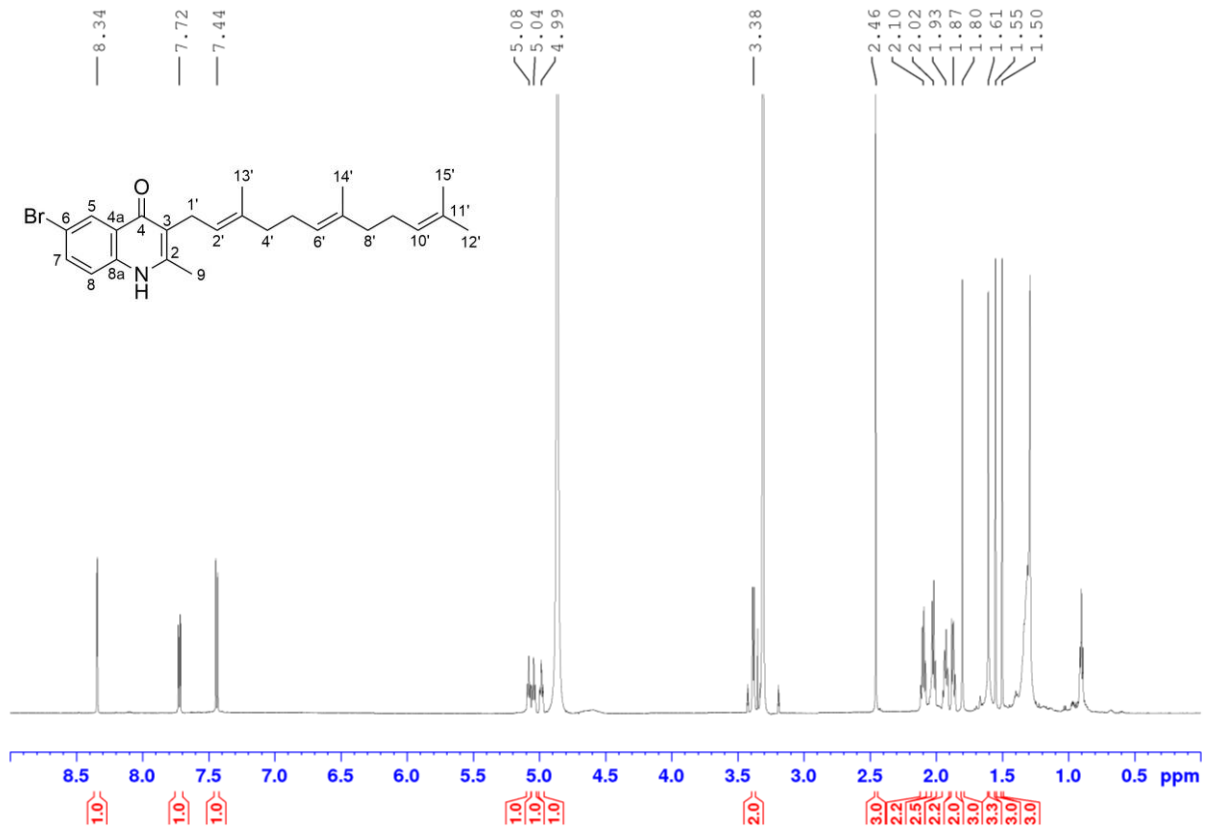

Figure S39. ^{1}H NMR spectrum (600 MHz, methanol-d_4) of **10**.

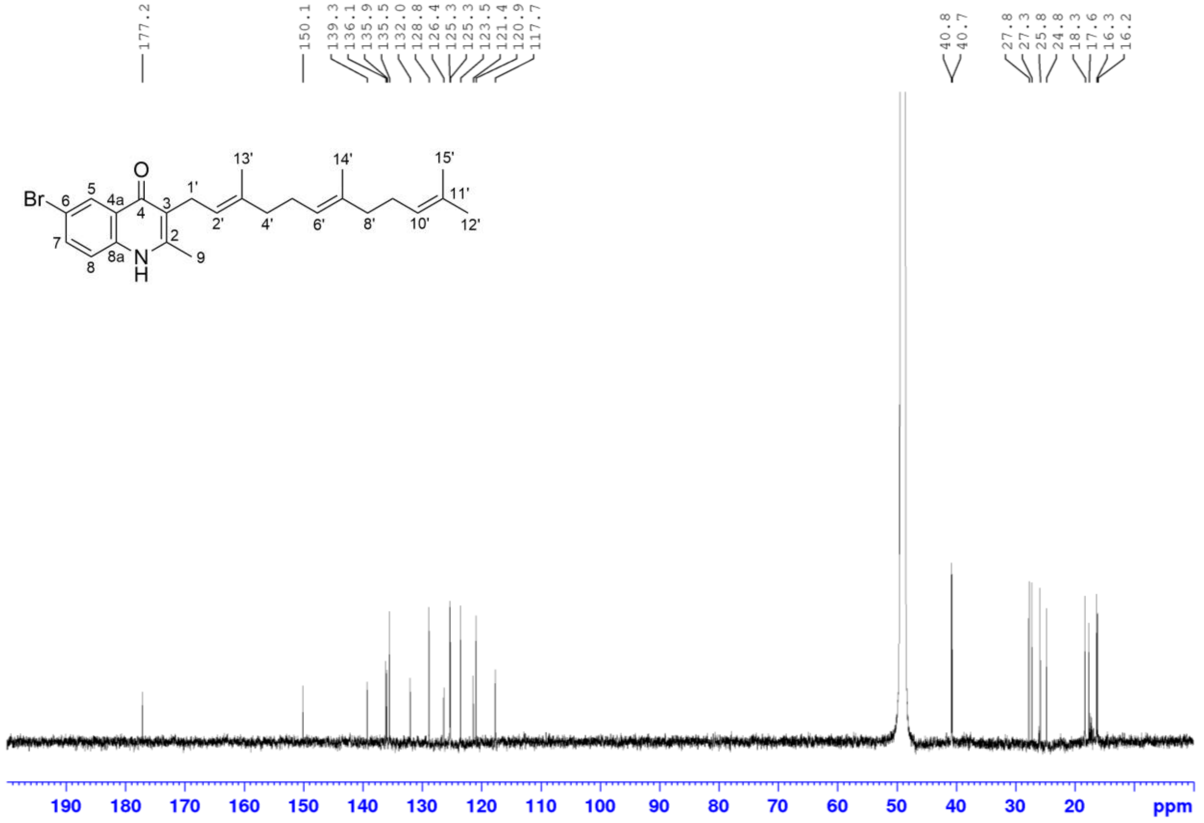

Figure S40. ^{1}H-decoupled ^{13}C NMR spectrum (150 MHz, methanol-d_4) of **10**.

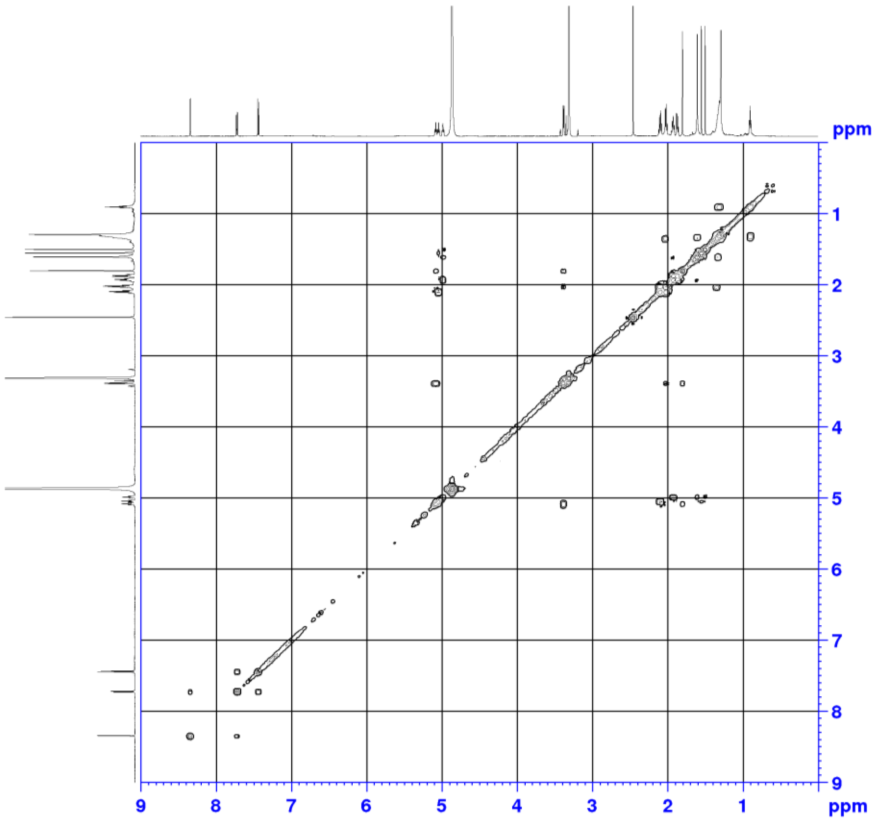

Figure S41. COSY spectrum (methanol-d_4) of **10**.

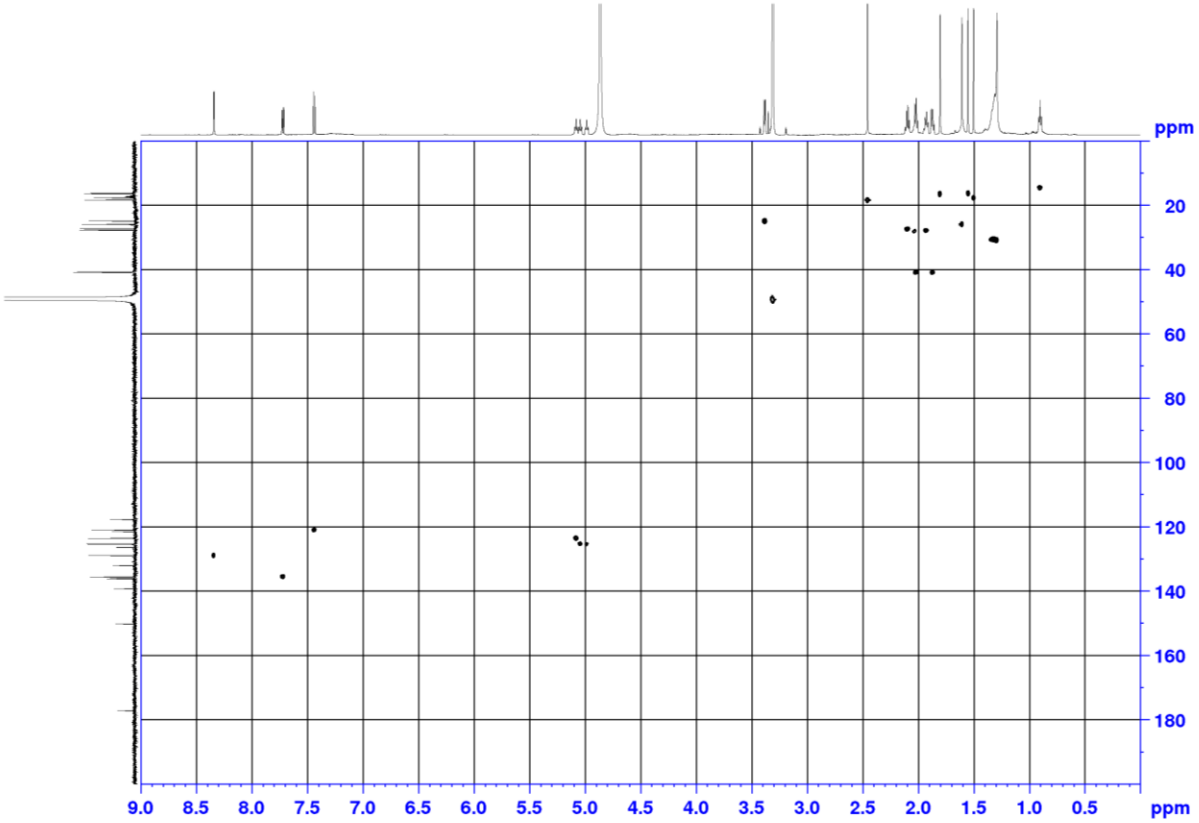

Figure S42. HSQC spectrum (methanol-d_4) of **10**.

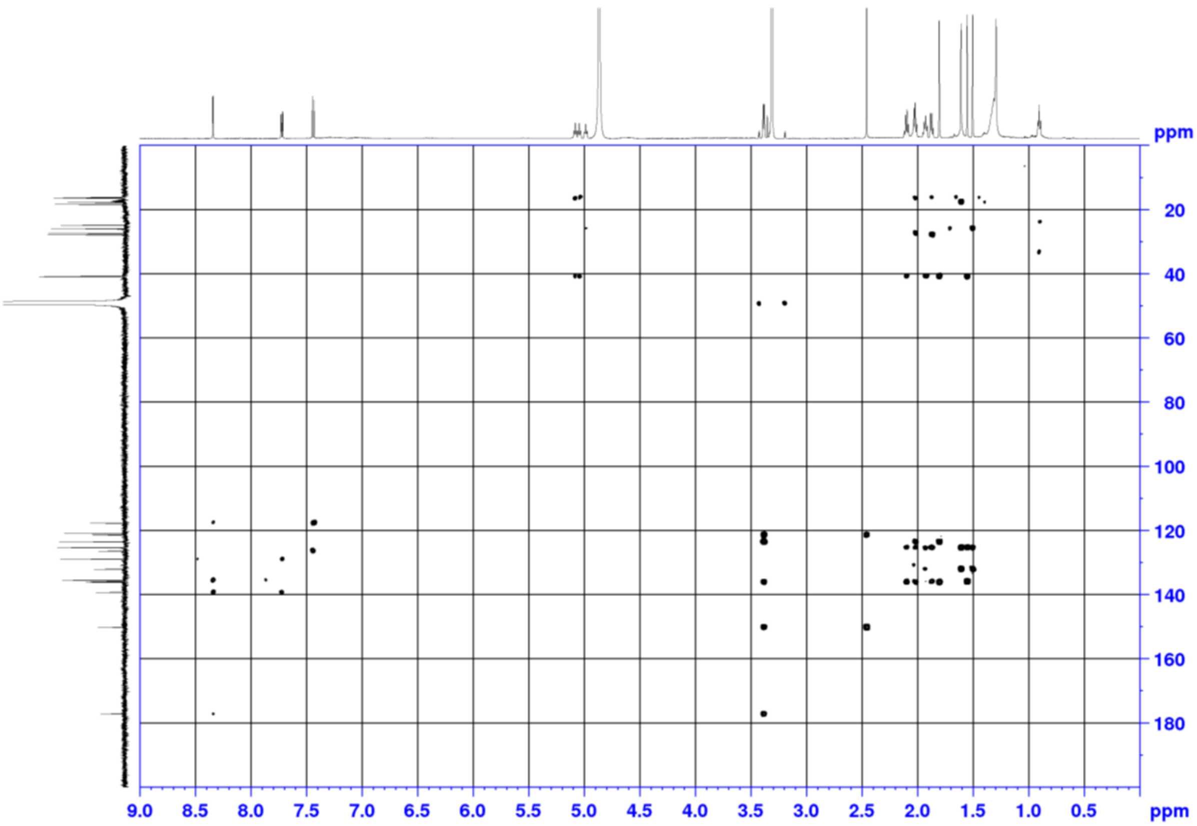

Figure S43. HMBC spectrum (methanol-d_4) of **10**.
